# The potential use of hydrogen in the extraction of phytochemicals from neglected and underutilized plant species for enhanced human nutrition

**DOI:** 10.3389/fnut.2026.1774917

**Published:** 2026-03-11

**Authors:** Emel Uçarer, Yasemin Arslan Hüdaverdi, Muhammed Allam Elnasanelkasim, Enes Kavrut, Solmaz Alkan, Kairat Bekbayev, Akerke Toleugazykyzy, Bakytzhan Bolkenov, Roza Bekbayeva, Abdyssemat Samadun, Duried Alwazeer

**Affiliations:** 1 Department of Nutrition and Dietetics, Faculty of Health Sciences, Igdir University, Igdir, Türkiye; 2 Department of Nutrition and Dietetics, Institute of Health Sciences, Gazi University, Ankara, Türkiye; 3 Department of Nutrition and Dietetics, Institute of Health Sciences, Istanbul Aydin University, Istanbul, Türkiye; 4 Department of Food Engineering, Faculty of Agriculture, Hatay Mustafa Kemal University, Hatay, Türkiye; 5 Tourism Faculty Gastronomy and Culinary Arts Department, Igdir University, Igdir, Türkiye; 6 Innovative Food Technologies Development, Application, and Research Center, Igdir University, Igdir, Türkiye; 7 Department of Nutrition and Dietetics, Institute of Health Sciences, Istanbul Medipol University, Istanbul, Türkiye; 8 Research School of Food Engineering, Shakarim University, Semey, Kazakhstan; 9 Institute of Engineering and Food Technology, S. Seifullin Kazakh Agrotechnical Research University, Astana, Kazakhstan; 10 Research Institute of Food Safety, Almaty Technological University, Almaty, Kazakhstan

**Keywords:** global nutrition, hydrogen extraction, neglected and underutilized plant species, phytochemicals, valorization

## Abstract

Neglected and underutilized plant species (NUS) are crops rich in bioactive phytochemicals that positively impact nutrition and health. Despite their resilience and nutritional benefits, NUS remain underexploited due to limited research funding, weak market demand, and postharvest issues. Identifying NUS rich in phenolic compounds, carotenoids, and micronutrients that can be recovered can facilitate their use across sectors such as food, pharmaceuticals, nutraceuticals, cosmetics, agrochemicals, and other industries. Incorporating NUS extracts into functional foods, supplements, and nutraceuticals can help address micronutrient deficiencies and promote sustainable food security. Promoting NUS valorization through advanced green technologies, such as hydrogen-based extraction, can enhance market appeal and reduce reliance on major crops. Various traditional and innovative techniques are used to extract phytochemicals from NUS. Recently, hydrogen extraction (H2Ext) has gained attention for its efficiency, sustainability, environmental friendliness, and cost-effectiveness. This review examines the potential of H2Ext to valorize NUS and recover phytochemicals, identifies research gaps and limitations, and emphasizes the strategic role of NUS and green technologies in global nutrition. It underscores the dual benefit: scientific progress via H2Ext and societal gains through increased nutritional security from underutilized biodiversity. H2Ext could reveal the hidden nutritional value of NUS by enhancing the release, stability, and chemical accessibility of phytochemicals from plant matrices.

## 1 Introduction

### 1.1 Background on human nutrition and phytochemicals

Human nutrition is not limited to essential macronutrients (carbohydrates, proteins, and fats) and micronutrients (minerals and vitamins). In addition to these main dietary components, bioactive compounds, including phytochemicals, zoochemicals, and microbial metabolites, play essential roles in the human diet. Among these bioactive compounds, phytochemicals have attracted attention for their diverse health benefits. Phytochemicals are bioactive compounds naturally present in plants, encompassing major classes such as phenolic compounds (including polyphenols and flavonoids), terpenoids (e.g., carotenoids), alkaloids, and sulfur-containing compounds like glucosinolates, many of which are associated with diverse health benefits. The health benefits of phytochemicals for humans include antioxidative and anti-inflammatory activities, as well as modulatory effects on metabolic, immune, and cellular signaling pathways; however, these represent only part of the wide range of protective and health-promoting functions associated with diets rich in phytochemical-containing plants (1, 2). Different phytochemicals have different health benefits. Polyphenols, including flavonoids, are phenolic compounds widely recognized for their strong antioxidant properties and their reported ability to modulate inflammatory processes. Flavonoids, which constitute a major subgroup of polyphenols, exhibit a broad spectrum of biological activities, including antioxidant and anti-inflammatory effects (3). Alkaloids are another important class of phytochemicals and are commonly associated with metabolic regulation and effects on the central nervous system, although their biological activities vary with structure and dose. In addition, many phytochemicals have been shown to possess antimicrobial, anticarcinogenic, and antidiabetic properties (4). Over the past two decades, increasing evidence has highlighted the significant role of phytochemicals in the prevention and management of chronic diseases. According to the World Health Organization, approximately 80% of the global population relies on plant-derived compounds for the treatment of disease. Furthermore, phytochemicals are increasingly applied in food preservation and in the development of functional foods (2, 5).

In addition to their health benefits, phytochemicals are extracted for use in the food and food additives, nutraceutical, pharmaceutical, and pesticide industries. However, the practical use of phytochemicals in the aforementioned industries depends on the efficiency of extraction methods. Traditional extraction methods often require large volumes of organic solvents, high energy consumption, and long processing times, which can adversely affect extraction yield, product quality, and environmental sustainability (6). As a result, growing attention has been given to the development of green extraction technologies. A range of green extraction methods, such as ultra-high-pressure extraction, aqueous extraction, enzymatic extraction, and air-explosion-assisted extraction, have been developed to reduce solvent use and improve extraction efficiency. Although these approaches aim to minimize the use of organic reagents, most still require small amounts of solvents or auxiliary agents to achieve satisfactory yields. In this context, hydrogen-assisted extraction has emerged as a promising alternative. Instead of completely eliminating organic solvents, this method reduces their use while enhancing mass transfer and cell disruption under relatively mild conditions. Compared with other green extraction techniques, hydrogen-assisted extraction may offer advantages such as shorter extraction times and better preservation of bioactive compounds (7). Although hydrogen is flammable, the dissolved hydrogen levels in water and organic solvents are very low, posing minimal safety concerns. The maximum dissolved hydrogen concentration in water and organic solvents is below 17 mg/L at STP conditions (8). Furthermore, the hydrogen extraction process is typically conducted in controlled environments under appropriate safety protocols, thereby reducing the risk of explosion and ensuring safe operation.

As the global population continues to grow, food security is becoming a pressing issue. With a projected population of 10 billion in 2050, humanity needs to address this issue (9). Neglected and underutilized plant species (NUS) hold significant promise in addressing the challenges of global nutritional security (10, 11). In recent years, NUS has started to gain recognition. However, barriers to the full utilization of NUS remain, including limited awareness, a lack of agronomic research, and postharvest and processing challenges. Additionally, when NUS targets phytochemicals, extraction is a significant challenge. Emerging green extraction technologies might help to overcome this challenge.

### 1.2 Importance of sustainable extraction technologies

Most phytochemicals are present in plant tissues at low concentrations; therefore, extraction methods are used to isolate them. Conventional extraction methods (e.g., maceration and Soxhlet extraction) often use organic solvents such as methanol, hexane, acetone, or ethanol. Although ethanol is a food-grade solvent, other ones, such as methanol, pose toxicity, and environmental concerns. On the other hand, these techniques may require high energy input, involve long processing times, and can cause thermal or hydrolytic degradation of heat-sensitive compounds, particularly in hydrodistillation due to prolonged exposure to boiling water (4, 12, 13). A contemporary review of green extraction methods emphasizes that non-polar, non-toxic solvents enable the environmentally friendly, consumer-safe extraction of phytochemicals. These approaches minimize the use of organic solvents, save time and energy, and increase the yield of valuable biomolecules. The transition to sustainable extraction technologies is increasingly important given the growing demand for safe and natural ingredients for food, nutraceuticals, and cosmetics (14, 15). As nutrition remains a pressing issue, the extraction of natural ingredients is increasingly essential for formulating functional foods, nutraceuticals, and clean-label products. The demand for bioactive compounds with proven health benefits continues to grow, driving the need for efficient and environmentally responsible extraction technologies (16).

### 1.3 Importance of neglected and underutilized species for nutritional security

Many NUSs have long been part of local food traditions, yet they remain largely overlooked in modern food production. These plants often contain a wide range of unusual phytochemicals—compounds such as uncommon antioxidants, distinctive secondary metabolites, and nutrient-dense components that are rarely found in today's dominant crops (17, 18). These biochemical characteristics make NUS particularly attractive for the development of functional foods, natural additives, and health-oriented food products (19). Beyond their nutritional value, many NUS are well adapted to marginal soils and to climate-stressed environments, enabling them to produce reliable yields under conditions in which conventional crops often fail. This resilience enhances their relevance for improving nutritional security, especially in regions vulnerable to climate variability and limited agricultural inputs (20). However, despite their recognized potential, NUS remain underexploited in global agriculture. Progress has been constrained by insufficient phytochemical characterization, limited agronomic research, weak value chains, and the lack of established markets (18).

Although many NUSs have been used in traditional diets and practices for generations, a large proportion of these plants have not been thoroughly evaluated from a toxicological standpoint. Moreover, many are not formally recognized by regulatory authorities such as the Codex Alimentarius Commission or JECFA as foods, pharmaceuticals, or approved sources of phytochemicals. In this review, traditional use is therefore viewed as a starting point that signals potential relevance, rather than as proof of safety. Accordingly, we do not assume that NUS are inherently safe; instead, we emphasize the need for well-designed toxicological studies, proper risk assessment, and regulatory validation before these species or their derived phytochemicals can be responsibly developed for food or health-related applications. Addressing these challenges will require targeted scientific studies to better document their nutritional and phytochemical composition, as well as improvements in processing and extraction technologies. In parallel, the development of sustainable commercialization pathways is necessary to integrate NUS into existing food systems. Together, these efforts could enable NUS to contribute more effectively to diet diversification, improved nutrition, and the resilience of future food systems.

### 1.4 Role of hydrogen as a green extraction enhancer

Hydrogen (H_2_) can act as a potent green extraction enhancer by solubilizing compounds, protecting sensitive molecules, and reducing oxidative degradation during extraction. Moreover, the utilization of hydrogen in the extraction makes the extraction more efficient, selective, and sustainable (21). From a sustainability perspective, hydrogen is considered a clean energy carrier because it produces no harmful emissions, with only water as a byproduct.

Hydrogen is increasingly being explored as a supporting component in green extraction technologies due to several inherent characteristics that make it highly compatible with sustainable processing. One of its most important features is its strong reducing ability, which allows hydrogen to counteract oxidative reactions that commonly damage sensitive plant compounds during extraction (22). Many phytochemicals—such as polyphenols, flavonoids, pigments, and aromatic compounds—are easily oxidized under heat or prolonged exposure to air. In a hydrogen-rich solvent, the extraction system becomes more chemically stable, reducing the likelihood of unwanted degradation. In addition, hydrogen's extremely small molecular size gives it remarkable mobility, enabling it to diffuse rapidly through plant tissues and solvents. This enhances its interaction with cellular structures and helps release valuable bioactive compounds more efficiently (21, 23).

**Figure 1:**
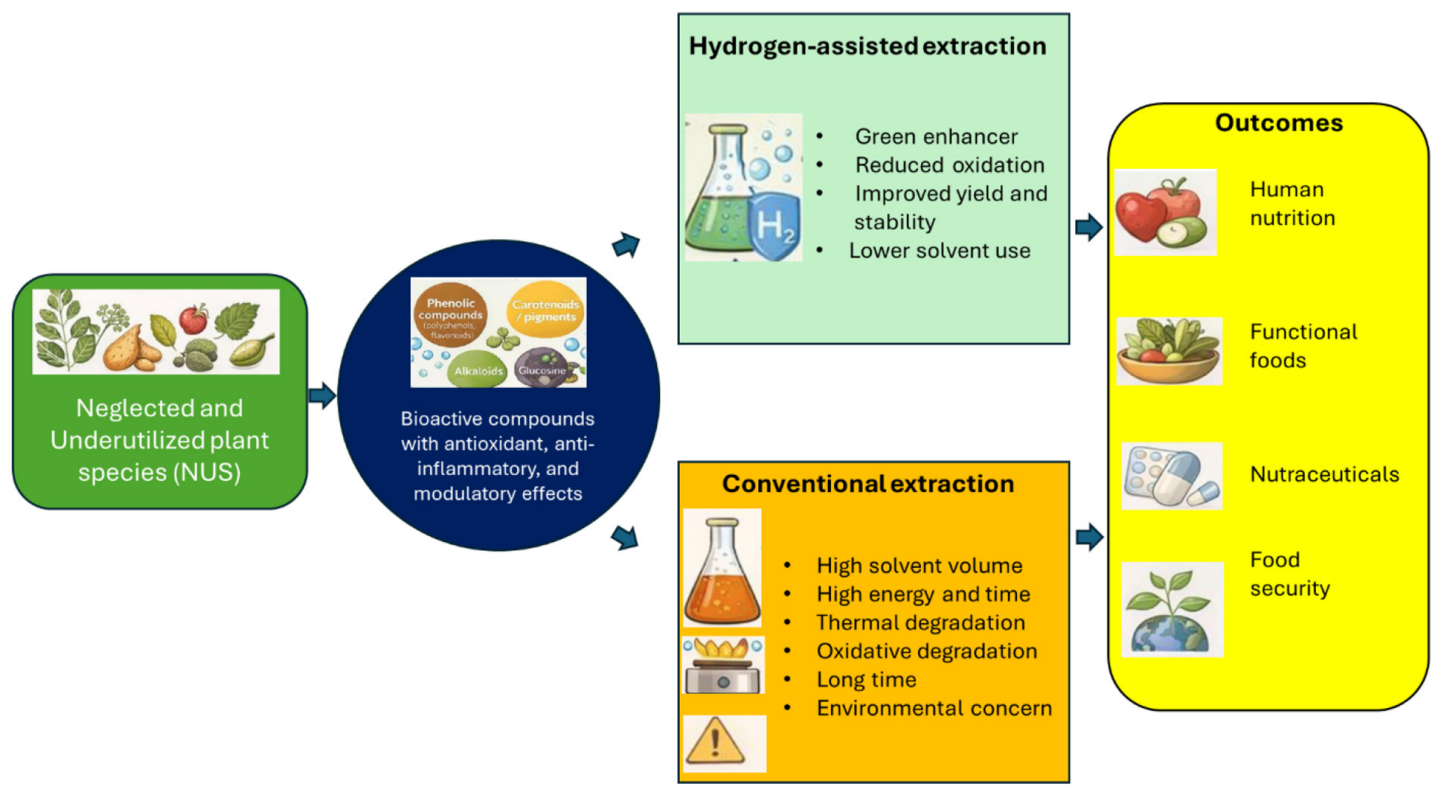
Some advantages of hydrogen-assisted extraction compared with conventional ones.

Another reason hydrogen attracts attention in green extraction research is its exceptional safety and environmental compatibility. Unlike many solvent modifiers or chemical additives, hydrogen does not introduce toxic residues or hazardous intermediates into the extraction system (24). It is naturally occurring and non-toxic; when used in energy or reaction processes, it produces only water as a by-product. These properties make hydrogen particularly appealing for applications in food, nutraceutical, and cosmetic industries, where extract purity and environmental safety are critical. The trend toward clean-label products and strict regulatory requirements further highlights the importance of adopting technologies that minimize chemical exposure while maintaining high extraction performance (Figure 1).

The integration of hydrogen into extraction workflows offers several practical benefits that extend beyond environmental motivations. By reducing oxidative stress and stabilizing delicate compounds, hydrogen helps maintain the natural bioactivity of complex plant extracts. This preservation of bioactivity is essential for industries focused on functional foods, herbal remedies, and bioactive-rich formulations. Additionally, hydrogen-assisted extraction has shown potential for improving overall extraction yields. Its high diffusivity promotes greater mass transfer, enabling valuable compounds to migrate into the solvent more effectively. This improved efficiency often translates into faster extraction cycles, better selectivity for target molecules, and fewer impurities co-extracted from plant material (21, 25).

A further advantage is the potential to lower the amount of organic solvent required. When hydrogen improves solubilization and enhances mass transfer, the extraction system can operate with milder conditions and reduced solvent volumes. This not only decreases production costs but also minimizes the environmental footprint associated with solvent manufacturing, use, and disposal. In some approaches, hydrogen can even support semi-solvent or low-solvent extraction, aligning with global efforts to replace traditional solvents with greener alternatives. Collectively, these benefits position hydrogen as a valuable innovation for next-generation sustainable extraction technologies, offering a pathway toward cleaner, more efficient, and environmentally friendly processing of natural products.

### 1.5 Objective and scope of the review

This review aims to summarize current knowledge on the application of hydrogen-based technologies for extracting phytochemicals from NUS, with a focus on their relevance to human nutrition. The review discusses why phytochemicals from NUS are nutritionally important, highlights the shortcomings of conventional extraction methods, and explores how hydrogen, due to its unique physicochemical properties, may enhance extraction efficiency, compound stability, and yield. At the same time, the review does not overlook the challenges associated with hydrogen-assisted extraction. Potential limitations include scalability issues, safety concerns, and the need for additional infrastructure relative to conventional methods. The flammable nature of hydrogen introduces safety concerns that must be carefully managed through appropriate equipment, process design, and regulatory compliance, particularly in industrial-scale applications. The review also assesses the current level of technological readiness of hydrogen-assisted extraction, identifies key knowledge gaps, and considers the conditions required for its safe and practical implementation. Overall, the scope of this work encompasses phytochemical diversity in NUS, emerging hydrogen-assisted extraction mechanisms, potential nutritional and sustainability benefits, and the technical, economic, and safety considerations necessary for the responsible adoption of such approaches in both research and commercial settings.

## 2 Overview of neglected and underutilized plant species

### 2.1 Definition and characteristics of neglected and underutilized plant species

NUSs encompass wild or cultivated plant species that were once popular, have been cultivated as local food sources by communities in their regions of origin for generations, and have adapted to specific agroecological conditions. Currently, these species are neglected by mainstream agriculture due to various agricultural, genetic, economic, social, and cultural reasons, primarily driven by the dominance of industrial monoculture-centered agrifood systems and a few staple crops (26, 27).

Talabi et al. (28) define orphan crops—a closely related term—as indigenous plant species cultivated by smallholder farmers under subsistence systems, characterized by limited scientific investment and restricted participation in trade. The terminology related to NUS is complex; researchers have introduced descriptors such as “neglected and underutilized species” (NUS or NUPs), “underutilized crops,” “orphan crops,” and “lost crops” (28, 29). Although these descriptors are used interchangeably, subtle differences exist among them—“orphan crops” particularly conveys the absence of institutional support and research interest, whereas the term “underutilized” refers to the gap between actual and potential use. The term “neglected and underutilized plant foods” (NUPFs) specifically focuses on sustainable applications and dietary diversification policies and is more frequently used in applied research contexts (29, 30).

NUS are resilient plants that are climate-resilient, require low inputs, have high nutritional value, and can grow even in degraded ecosystems (27, 31). Thanks to these features, NUS are attracting increasing attention as climate-adaptive nutritious food options due to critical attributes such as reducing dependence on staple foods, preserving cultural dietary diversity, adapting to climate change, conserving agricultural biodiversity, and contributing to the overall sustainability and resilience of food systems (27, 32, 33). Furthermore, NUS possess significant potential for the realization of Sustainable Development Goals (SDGs), particularly No Poverty (SDG 1), Zero Hunger (SDG 2), Good Health and Wellbeing (SDG 3), decent work and economic growth (SDG 8), responsible consumption and production (SDG 12), climate change adaptation and mitigation (SDG 13), and Life on Land (SDG 15) (34).

**Figure 2:**
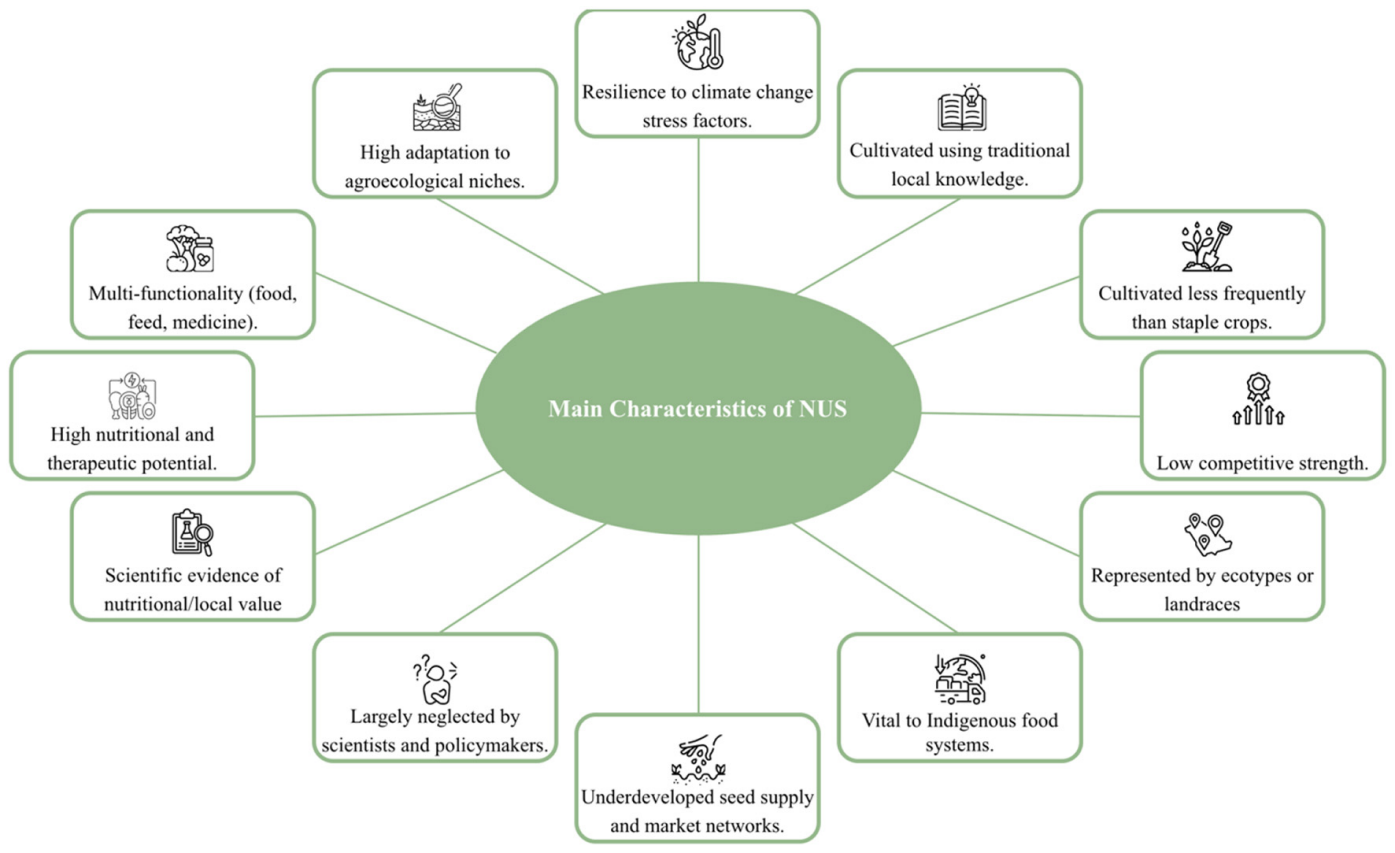
The characteristic features of NUS.

The characteristic features of NUS are illustrated in Figure 2 (35, 36, 198).

In addition to terms such as indigenous and traditional food crops, orphan crops, and minor crops, NUS are now also referred to as *Future Smart Food*. However, to be defined as Future Smart Food (FSF), NUS are required to present four core attributes simultaneously: nutritional richness (improving nutrition), climate resilience (increasing environmental sustainability), local availability/adaptability, and cost-effectiveness (income generation, reducing workload) (37).

NUS includes many food groups such as cereals, legumes, vegetables, tree products, fruits, oilseeds, roots, and tubers (Table 1) (38–41, 198).

**Table 1:** Food groups and examples of neglected and underutilized species (NUS).

**Food groups**	**Examples of neglected and underutilized species (NUS)**
Cereals	Einkorn (*Triticum monococcum*), emmer (*T. dicoccon*), tef (*Eragrostis tef*), fonio (*Digitaria exilis*), sara (*Zea mays*), papa andina (*Solanum tuberosum* L.), kodo millet (*Paspalum scrobiculatum*), little millet (*Panicum sumatrense*), amaranth (*Amaranthus caudatus*), buckwheat (*Fagopyrum* spp.), sorghum (*Sorghum bicolor*)
Fruits and nuts	Maya nut (*Brosimum alicastrum*), breadfruit (*Artocarpus altilis*), baobab (*Adansonia digitata*), jujube (*Ziziphus mauritiana*), cherimoya (*Annona cherimola*), cape gooseberry (*Physalis peruviana*), naranjilla (*Solanum quitoense*), noni (*Morinda citrifolia*), marula (*Sclerocarya birrea*), tamarind (*Tamarindus indica*), annona (*Annona* spp.), safou (*Dacryodes edulis*), mangosteen (*Garcinia mangostana*), monkey orange (*Strychnos cocculoides*), camu camu (*Myrciaria dubia*), Brazil nut (*Bertholletia excelsa*), egg nut (*Couepia longipendula*), quince (*Cydonia oblonga*)
Legumes	mung bean (*Vigna radiata*), adzuki bean (*V. angularis*), ricebean (*V. umbellata*), cowpea (*Vigna unguiculata*), bambara groundnut (*Vigna subterranea*), jack bean (*Canavalia ensiformis*), grasspea (*Lathyrus sativus*), lablab bean (*Lablab purpureus*), pigeon pea (*Cajanus cajan*), Kersting's groundnut (*Macrotyloma geocarpum*)
Vegetables	African eggplant (*Solanum aethiopicum*), spider plant (Cleome gynandra), leaf amaranth (*Amaranthus* spp.), brassica (*Brassica rapa* varieties), African/black nightshade (*Solanum scabrum/nigrum*), okra (*Abelmoschus esculentus*), Ceylon spinach (*Basella rubra*)
Roots and tubers	Yams (*Dioscorea* spp.), yacon (*Smallanthus sonchifolius*), cassava (*Manihot esculenta*), ulluco (*Ullucus tuberosus*), taro (*Colocasia esculenta*), arracacha (*Arracacia xanthorriza*), American yam bean (*Pachyrhizus* spp.), maca (*Lepidium meyenii*), oca (*Oxalis tuberosa*), sweet potato (*Ipomoea batatas*)
Medicinal and aromatic plants	Moringa (*Moringa oleifera* and *Moringa stenopetala*), tannia (*Xanthosoma sagittifolium*), culantro (*Eryngium foetidum*), pink pepper (*Schinus molle*), roselle (*Hibiscus sabdariffa*), cat's claw (*Uncaria tomentosa*), white willow (*Salix alba* L.), barbados gooseberry (*Pereskia aculeata*), false roselle (*Hibiscus acetosella*), andiroba (*Carapa guianensis*), clove basil (*Ocimum gratissimum*), fever tea (*Lippia javanica*), copaiba (*Copaifera langsdorffii*), purslane (*Portulaca oleracea*)

NUS demonstrates adaptation to challenging environmental conditions, including poor soils, water stress, and extreme climates, making it highly valuable for cultivation in resource-limited settings. Thus, they are critical to climate change and the need for resilient agricultural systems (28). Compared with popular crops, NUS receive limited research investment, have insufficient agronomic documentation, and undergo restricted breeding efforts, resulting in substantial knowledge gaps regarding their cultivation requirements, yield potential, and possibilities for genetic improvement (42). NUS often possess strong local and ethnobotanical significance and are embedded within traditional food cultures and indigenous knowledge systems; however, they lack pathways for international commercialization (29, 43). Finally, NUS are known to have superior nutritional profiles, with higher concentrations of bioactive compounds than conventional staple crops, making them promising candidates for dietary enrichment and functional food development (44).

### 2.2 Geographical distribution and biodiversity hotspots

NUSs reflect agricultural biodiversity shaped by thousands of human–environment interactions. Comprehensive global inventories remain limited. Recent studies highlight important NUS examples across tropical lowlands, mountain regions, Mediterranean ecosystems, and South Asian smallholder landscapes (42, 45).

South Asian agricultural landscapes—from the Indo-Gangetic plains to coastal zones—contain numerous locally important NUS within small-scale farming systems. Recent case studies from Sri Lanka and the broader South Asian context outline pathways for integrating these species into modern diets and cropping systems, emphasizing their roles in enhancing resilience and dietary diversity (43). Sow thistle (*Sonchus oleraceus* L.), grass pea (*Lathyrus sativus* L.), eggplant (*Solanum melongena* L.), and snake melons (*Cucumis melo* var. *flexuosus* (L.) Naud.) are among the significant NUS examples widely utilized within the traditional agricultural systems and dietary frameworks of South Asia (46). The Himalayan region—particularly the northern Himalayas spanning India, Nepal, and Bhutan—constitutes a significant biodiversity hotspot for underutilized plant species. Bachheti et al. (45) document a wide variety of wild fruits used by numerous local communities, including *Ficus auriculata, Rubus ellipticus, Myrica esculenta, Berberis asiatica*, which hold both nutritional and medicinal roles in traditional systems. These mountain ecosystems, characterized by altitudinal gradients and microhabitat diversity, have preserved the evolution and persistence of diverse plant genetic resources that remain largely unexploited in formal agricultural and food systems (45). South Asia possesses an exceptionally rich heritage of medicinal plant resources. The Himalayan region, in particular, constitutes one of the world's most significant reservoirs for medicinal plant diversity. Within this landscape, species such as white Himalayan lily *(Lilium polyphyllum*), muskroot *(Nardostachys jatamansi*), purslane (*Portulaca oleracea*), and chekurmanis (*Sauropus androgynus*) stand out as significant examples of NUS (47).

The African continent, which is reported to harbor the developmental potential for over 40,000 plant species, stands as one of the world's most biodiverse yet least explored regions with respect to NUS. Crops such as the bambara groundnut (*Vigna subterranea* (L.) Verdc.), soursop (*Annona muricata* L.), and fonio (*Digitaria exilis* (Kippist) Stapf) are prominent examples of NUS originating from this region, where they remain actively integrated into traditional medicine and indigenous food systems. Furthermore, the distribution of NUS across Africa exhibits distinct variations dictated by specific ecological zones (48, 49). In West Africa, particularly in countries such as Nigeria, the African walnut (*Tetracarpidium conophorum*) and African breadfruit (*Treculia africana*) are prominent indigenous species (50). Furthermore, fonio millets (*Digitaria* spp.), which are highly adapted to the region's arid climate, and Kersting's groundnut (*Macrotyloma geocarpum*) represent significant crops within the West African agricultural landscape (51, 52). In East Africa, specifically in Ethiopia, enset (*Ensete ventricosum*), commonly known as the “false banana,” serves as a vital staple food source and is recognized as a strategic species for its environmental benefits, such as preventing soil erosion (53). Pearl millet (*Pennisetum glaucum*), sorghum (*Sorghum bicolor* L.), and cowpea (*Vigna unguiculata*) are among the representative examples of NUS grown in South Africa. Additionally, species such as the bambara groundnut (*Vigna subterranea* L.), marula (*Sclerocarya birrea*), and spider plant (*Cleome gynandra*) are recognized as strategic species indigenous to sub-Saharan Africa, including South Africa, and are valued for their exceptional adaptation to local ecosystems and traditional nutritional significance (54).

Following Asia and Africa, South America ranks as the most species-rich region in terms of NUS diversity (18). The South American landscape is structured into diverse biodiversity hotspots, primarily the high-altitude Andean range and the lowland Mesopotamia basin. The Andes constitute a premier center for *Capsicum* speciation, hosting significant wild relatives like *Capsicum baccatum* and *Capsicum pubescens*, along with resilient tubers such as mashua (*Tropaeolum tuberosum*), arracacha (*Arracacia xanthorrhiza*), and ulluco (*Ullucus tuberosus*). In contrast, the Mesopotamia region—encompassing Northeastern Argentina, Uruguay, and parts of Southern Brazil—serves as a vital reservoir for neglected medicinal and aromatic plants, including sticky nightshade (*Solanum sisymbriifolium*), pink pepper (*Schinus terebinthifolius*), and clove basil *(Ocimum gratissimum* L.) (18). In addition to these regions, sacha inchi (*Plukenetia volubilis*), native to the Amazon region and once an important crop of pre-Columbian societies, has only recently received renewed scientific attention due to its exceptional oilseed characteristics and nutritional profile (42). Its adaptation to humid tropical conditions and integration into traditional agroforestry systems underscore NUS diversity in this region. Furthermore, the Amazon Basin is a biodiversity center for species such as camu-camu (*Myrciaria dubia*), which exhibits extraordinary vitamin C levels and antioxidant capacity (55).

Mediterranean and temperate European regions host distinctive NUS assemblages among native forest fruit trees and shrubs. Karapatzak et al. (44) identify Greek native species such as *Amelanchier ovalis, Cornus mas, Rosa canina*, and *Sambucus nigra* as NUS with considerable antioxidant potential and opportunities for sustainable use. Adapted to Mediterranean climate regimes and historically integrated into rural economies, these species represent genetically rich resources for horticultural development and nutraceutical applications (44). Although regional examples demonstrate NUS distribution, the literature lacks standardized global comparisons or validated biodiversity hotspot rankings based on NUS richness. Evidence is regionally detailed, yet a systematic global assessment for hotspot prioritization is absent (42, 45). This gap reflects broader challenges in NUS research, including inconsistent documentation, limited taxonomic resolution, and the absence of coordinated international inventories.

### 2.3 Nutritional and phytochemical potential of NUS

NUSs possess both significant nutritional and functional value. They offer sustainable sources rich in essential macronutrients such as protein, carbohydrates, essential fatty acids, and dietary fiber, as well as vital micronutrients such as vitamins, calcium, zinc, and iron (18, 56, 57) (Table 2). Substantial evidence indicates that NUS presents a viable solution for addressing nutritional deficiencies by increasing dietary diversity, complementing staple foods (such as cereals typically poor in lysine), and strengthening local traditional foods (18, 25, 56). This nutritional superiority positions NUS as a critical resource for combating malnutrition, alleviating dietary monotony, and addressing the rising prevalence of diet-related chronic diseases (28, 45).

**Table 2:** Nutritional and phytochemical profile of selected neglected and underutilized species (NUS).

**Category**	**Species**	**Nutritional profile**	**Phytochemicals**	**References**
Legumes	bambara groundnut (*Vigna subterranea*)	High-quality protein	Flavonoid (catechins, epicatechins, proanthocyanidins)	(58)
	rice bean (*Vigna umbellata*)	High protein, Minerals (iron and zinc), vitamins (ascorbic acid, niacin)	Flavonoid (isoquercitrin, procyanidin B1, rutin, taxifolin, and catechin)	(59, 60)
Cereals	fonio (*Digitaria exilis*)	Starch, gluten-free, high concentration of methionine	Phenolic acids (Ferulic acid, protocatechuic acid, caffeic acid), alkaloids	(61)
	buckwheat (*Fagopyrum* spp.)	Gluten-free, high protein, high dietary fiber	Flavonoids (rutin, quercetin, catechin), Phenolic acids (gallic acid, ferulic acid, caffeic acid)	(62)
Roots and tubers	yams (*Dioscorea* spp.)	Starch, vitamin C, vitamin B1	Flavonoids (quercetin), tannin, saponin (diosgenin), alkaloids	(63, 64)
	taro (*Colocasia esculenta*)	High resistant starch. Protein, dietary fiber, and micronutrients in leaves	Flavonoids (quercetin, catechin), phenolic acids (gallic acid, benzoic acid, caffeic acid) alkaloids	(65, 66)
Fruits	baobab *(Adansonia digitata)*	Vitamin C, dietary fiber, potassium, and phosphorus	Flavonoid (epicatechin-3-gallate, proanthocyanidins, procyanidin B2), tannin	(67)
	camu camu (*Myrciaria dubia*)	Vitamin C	Flavonoids (quercetin, catechins, anthocyanins), tannins, phenolic acids (gallic acid and ellagic acid)	(68)
Leafy vegetables	amarant (*Amaranthus* spp.)	Dietary fiber, carotenoids, vitamin C	Flavonoid (quercetin, iso-quercetin, rutin, kaempferol, catechin, naringenin)	(69)
Medicinal and aromatic plants	*water spinach* (*Ipomoea aquatica*)	Alpha-linolenic acid, minerals (potassium and calcium)	Flavonoid (quercetin) phenolic acids (gallic acid)	(70)
	moringa (*Moringa oleifera*)	Leaves: high protein, dietary fiber Seeds: protein, dietary fiber, fatty acids	Leaves: flavonoids (quercetin, apigenin, kaempferol, and luteolin), phenolic acids, alkaloids, glucosinolates Seeds: glucosinolates	(71, 72)
	clove basil (*Ocimum gratissimum*) leaves	Dietary fiber, minerals (calcium, magnesium, phosphorus)	Flavonoids (epicatechin, quercetin, rutin, apigenin, kaempferol), tannin essential oils, saponins, alkaloids	(73, 74)

Through these roles, they have the potential to improve the micronutrient content in the diets of millions of people worldwide. Beyond their nutritional value, they provide numerous health benefits thanks to the phytochemicals found in these products. These components can exhibit antioxidant, anti-inflammatory, and anticarcinogenic properties (46). Furthermore, the fact that many NUS, such as Buckwheat, are naturally gluten-free further increases their importance in maintaining health and preventing diet-related health issues (75, 76). NUSs have been shown to contain a broad array of bioactive compounds with health-enhancing properties. Karapatzak et al. (44) conducted comprehensive assessments of indigenous Greek wild fruits, documenting notable free radical scavenging activity and substantial antioxidant potential across various genotypes of Amelanchier, Cornus, Rosa, and Sambucus. These results indicate considerable prospects for the development of functional foods and nutraceutical products derived from Mediterranean NUS (44). In a similar vein, Research on wild fruits from the northern Himalayan region has identified significant concentrations of macronutrients and micronutrients, alongside a diverse array of phytochemicals categorized as phenolic compounds (including flavonoids and tannins), isoprenoids (including saponins), and nitrogen-containing compounds (including alkaloids) (45). The health implications associated with these phytochemical profiles are substantial. Extracts and isolated compounds derived from various NUS have demonstrated an extensive range of biological activities in laboratory and preclinical investigations, encompassing antioxidant, anti-inflammatory, antimicrobial, antipyretic, analgesic, antiplasmodic, antinociceptive, antidiabetic, and anticancer effects (44, 45). While many of these findings necessitate confirmation through clinical trials, they establish a scientific foundation for the traditional medicinal applications of these species and suggest promising future roles in preventive health strategies.

### 2.4 Current barriers to utilization and commercialization

Although there are more than 30,000 plant varieties worldwide, only about 7,000 of them are edible (77). Today, while the vast majority of an individual's diet consists of 10–12 varieties, more than half of our energy needs are derived from wheat, maize, and rice (201). Since the Green Revolution, the focus on these major species to meet human nutritional needs has increased their popularity and decreased the utilization of numerous other species (27). This concentration has overshadowed the broader value of NUS; yet, its potential extends well beyond dietary contributions to include the extraction of bioactive compounds for natural pesticides and medicinal formulations. However, the inherent presence of anti-nutritional factors (ANFs)—which may impede nutrient absorption or pose health risks if consumed in high concentrations—necessitates rigorous safety protocols for their industrial integration (78). Despite this vast and multifaceted potential, there are interrelated systemic barriers to the utilization and commercialization of NUS in global food systems.

Negative assumptions about NUS and the stigmatization of these species as “famine food” or “poor man's food” adversely affect their production and marketing (79). Additionally, limited knowledge of the nutritional content of NUS, shifts in dietary habits due to changing lifestyles, and insufficient guidance on how to prepare these species are associated with the decline in their use over time (48). Inadequate investment in the production, processing, and storage of these products, and their inability to secure a place in market chains due to competition with other major products, are among the economic barriers facing NUS (28, 80). Furthermore, the lack of financing for necessary research, development, and marketing support for NUS, due to government and policy prioritization of major crops such as rice and wheat, is another limiting factor for NUS utilization. Additionally, the failure to establish connections between policymakers, national programs, research institutes, and farmers contributes to the neglect of NUS (81). These barriers are summarized in Table 3 below under the subheadings of social, economic, environmental, agronomic, political, and scientific (27, 46, 82, 83).

**Table 3:** Summary of current barriers to the utilization and commercialization of neglected and underutilized species (NUS).

Social	• Unfamiliar taste of NUS and lack of knowledge on how to prepare them • Farmers prefer new and improved varieties over traditional crops • Changes in dietary habits brought about by urbanization • Decline in knowledge regarding traditional and local species • Low awareness regarding the nutritional value of landraces • Negative assumptions and low status perception regarding some local and traditional foods • Migration of farm labor to urban areas
Economic	• Changes in land use • Lack of market infrastructure and low commercial value of NUS • Difficulty for NUS to compete with other crops • Lack of incentives for farmers to cultivate NUS • Inefficiencies in the production, processing, and storage of these crops
Environmental	• Genetic erosion of NUS due to causes such as drought, fires, pests, diseases, overgrazing, and deforestation • Environmental pollution • Ecosystem degradation and climate change • Overexploitation of wild resources
Agronomic	• Lack of seed supply systems and propagation materials • Presence of toxic and allergenic compounds • Insufficiency of trained personnel • Excessive use of pesticides, fertilizers, and other agrochemicals
Political	• Failure of national and local governments to prioritize the conservation and use of NUS • Insufficiency of governments in supporting scientific research on NUS • Lack of information on characterization, breeding, and evaluation • Absence of laws, policies, projects, national programs, and strategies • Lack of integration between conservation and utilization programs
Scientific	• Insufficient empirical evidence on the nutritional and health-related benefits of NUS. • Limited scientific data on the chemical constituents and toxicological safety of NUS. • A lack of formal recognition as safe food sources or phytochemical precursors by international bodies such as the FAO, WHO, or JECFA. • Deficient cross-sectoral alignment (agriculture/health) obstructing the dissemination of nutritional and safety evidence.

Case studies show that the successful development of NUS requires context-specific approaches that draw on local knowledge and directly target the most pressing obstacles (43). Even so, existing research highlights the absence of robust policy models and practical implementation frameworks, revealing a clear gap between recognizing the value of NUS and translating this recognition into concrete action (30). The challenges surrounding the use and commercialization of NUS are intertwined and multifaceted, making coordinated efforts among researchers, policymakers, market actors, and civil society essential. Addressing these barriers goes beyond technical fixes and brings forward broader questions about the direction of agricultural research, the governance of food systems, and how modern societies value agrobiodiversity (28, 29).

Inadequate acquisition and propagation of NUS seeds have led farmers to rely on fewer species and to reduce crop production. Moreover, it is thought that the use of chemicals and pollutants introduced by the modern world will contaminate the soil and harm NUS (84). Although NUS is more resilient than other major crops, it is also affected by temperature, precipitation, and climate, as well as by ecosystem changes. The possibility of preference shifting toward more resilient species under changing environmental conditions poses a threat to NUS in the coming years, which already faces the risk of genetic erosion (27).

## 3 Phytochemicals from neglected and underutilized plant species

**Figure 3:**
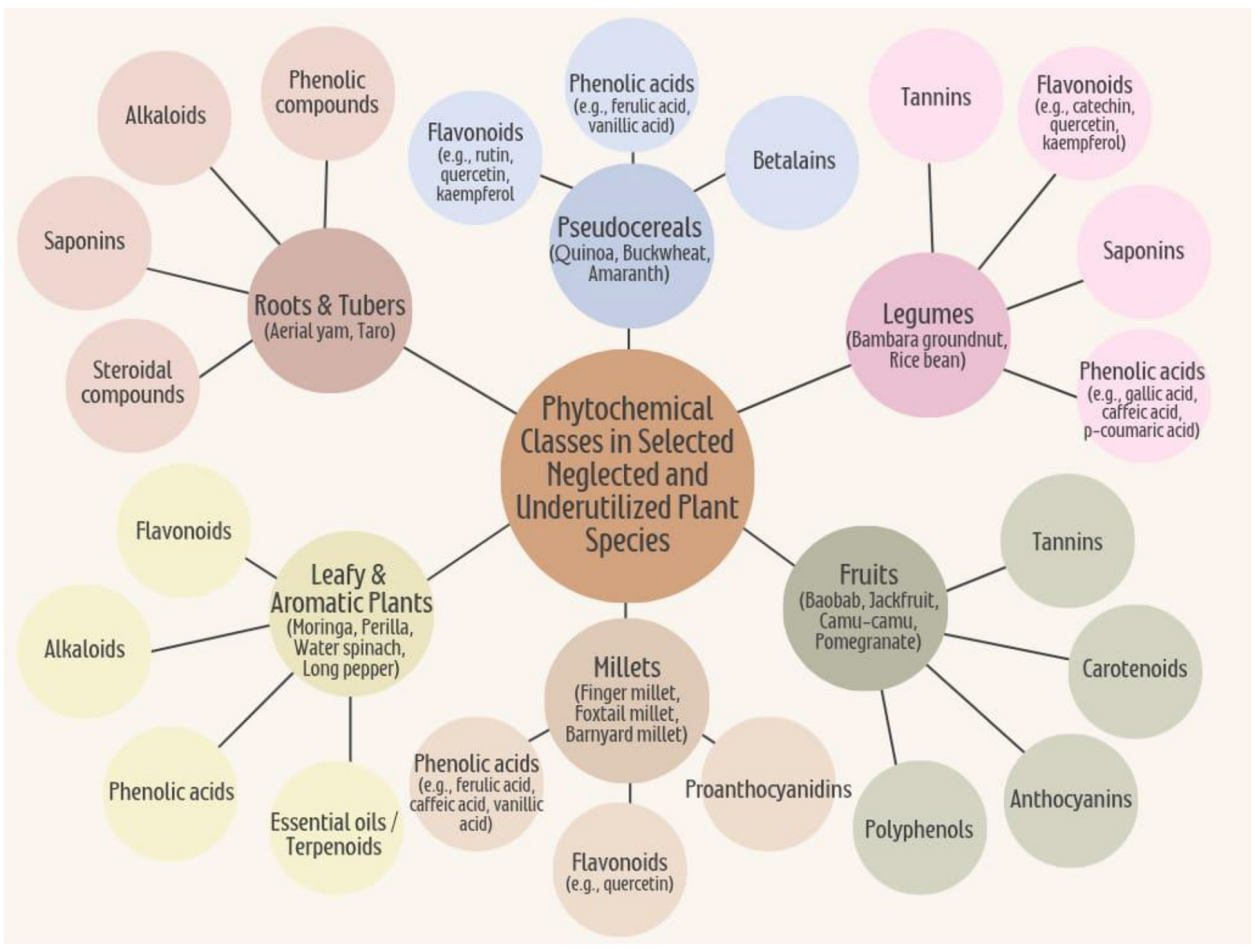
Phytochemicals found in neglected and underutilized plant species (NUS).

Phytochemicals consist of three major groups: (i) isoprenoids (terpenoids), (ii) phenolic compounds, and (iii) nitrogen-containing compounds. Phenolic compounds encompass polyphenols, a broad category that includes flavonoids, phenolic acids, tannins, and other related structures. Alkaloids are classified as nitrogen-containing compounds (Figure 3).

NUSs are rich in essential amino acids, vitamins, minerals, and bioactive compounds. Their nutritional content can help alleviate malnutrition, especially in impoverished communities. Therefore, many global organizations prioritize the assessment and improvement of NUS (85). Ingesting bioactive compounds supports certain physiological effects. Using NUS in conjunction with conventional medical treatments for various health problems may offer advantages in managing side effects, improving health, and preventing disease (86). The major bioactive components of some selected NUS samples are shown in Table 4.

**Table 4:** The major bioactive components of some selected neglected and underutilized species (NUS).

**Common name/Scientific name**	**Major bioactive compounds**	**References**
Bambara groundnut (*Vigna subterranea*)	anthocyanin, catechin, quercetin, kaempferol, rutin, myricetin, luteolin, chlorogenic acid, p-coumaric acid, ellagic acid, gallic acid, salicylic acid, caffeic acid and their derivatives, t-ferulic acid, quinic acid, medioresinol	(87)
Rice bean (*Vigna umbellata*)	catechin, epicatechin, p-coumaric acid, ferulic acid, vitexin, isovitexin, sinapic acid, and quercetin	(88)
Finger millet (*Eleusine coracana*)	quercetin, protocatechuic, vanillic acid, p-hydroxybenzoic acid, syringic acid, trans-cinnamic acid, p-coumaric acid, sinapic acid, ferulic acid, gallic acid	(89)
Foxtail millet (*Setaria italica*)	ferulic acid, chlorogenic acid, caffeic acid, p-coumaric acid, syringic acids	(89)
Barnyard millet (*Echinochloa crusgall*)	gallic acid, p-hydroxybenzoic acid, vanillic acid, caffeic acid, chlorogenic acid, ferulic acid, p-coumaric acid	(89)
Quinoa (*Chenopodium quinoa)*	quercetin, kaempferol, myricetin, isorhamnetin, ferulic acid-4-glucoside, betanin	(90)
Buckwheat (*Fagopyrum* spp.)	rutin, p–hydroxyl benzoic syringic acid, vanillic acid, gallic acid, protocatechuic acid, ferulic acid p –coumaric acid	(90)
Amaranth (*Amaranthus* spp.)	isoquercetin, rutin, nicotiflorine, amaranthine, betacyanin, zeaxanthin, lutein, β-carotene	(90)
Aerial Yam (*Dioscorea bulbifera*)	diosgenin, dioscin, diosbulbin, dioscorin, myricetin, quercetin, palmitic acid, rutin, luteolin, kaempferol, phytol, kaempferide, spiroconazol, flavanthrinin, dioscorine, diosbulbin	(91)
Taro (*Colocasia esculenta*)	astilbin, b-sitosterol, caffeic acid, isoorientin, orientin, rutin, stigmasterol, vicenin	(92)
Baobab (*Adansonia digitata*)	epigallocatechin gallate, procyanidin, gallic acid, proanthocyanidin, procyanidin, epicatechin, phytic acid	(67)
Jackfruit (*Artocarpus heterophyllus*)	artocarpesin, miricetin, benzyl (e)-ferulate, caffeic acid, chlorogenic acid, coumaroylquinic acid, ferulic acid, *p*-coumaric acid, and *trans*-3-(trifluoromethyl) cinnamic acid, octadecyl ester, all-trans-lutein, all-trans-β-carotene, all-trans-neoxanthin, 9-cis-neoxanthin, and 9-cis-vio-laxanthin	(93)
Camu camu (*Myrciaria dubia*)	gallic acid, ellagic acid, syringic acid, trans-resveratrol, ellagic acid, quercetin, myricetin, cyanidine-3-glucoside, myrciarone, rhodomyrtone, grandinin, castalagin, methylvescalagin, stachyurin, zeaxanthin, luteoxanthin, neoxanthin, violaxanthin, lutein, β-carotene, globulol, linalool, myrcene, spathulenol, limonene, betulinic acid	(94)
Water spinach (*Ipomoea aquatica*)	chlorogenic acid, *p*-coumaric acid, *trans*-ferulic acid, *trans*-cinnamic acid, epicatechin, quercetin, catechin, rutin hydrate, gallic acid, vanillic acid, vanillin	(95)
Moringa (*Moringa oleifera*) (various parts)	glucomoringin, glucosides, malonyl glucosides, rutinosides, quercetin, myricetin, epicatechin, rutin, caffeic acid, chlorogenic acid, coumaric acid, gallic acid, ellagic acid, tannins, glucosinolates, isothiocyanates, β-carotene, niazidin, niazicin, niazinin, vincosamide, trigonelline, moringinine, terpenes	(96)
Perilla (*Perilla frutescens*)	rosmarinic acid, rosmarinic acid-3-*O*-glucoside, caffeic acid, caffeic acid-3-*O*-glucoside, ferulic acid, catechin, apigenin, apigenin 7-*o*-glucuronide, apigenin 7-*O*-diglucuronide, luteolin, luteolin 7-*O*-glucuronide, luteolin 7-*O*-diglucuronide, scutellarein, scutellarein 7-*O*-glucuronide, scutellarein 7-*O*-diglucuronide, shisonin, malonylshisonin, cyanidin 3-*O*-caffeoylglucoside-5-*O*-glucoside, cyanidin 3-*O*-caffeoylglucoside-5-*O*-malonylglucoside, α-tocopherol, β-tocopherol	(97)
Long pepper (*Piper longum*)	piperine, piperlongumine, piperidine, palmitic acid, linoleic acid	(98)

Consumption of legumes from NUS has been associated with various health benefits due to the rich dietary fibers, essential amino acids, vitamins, polyunsaturated fatty acids (PUFAs), minerals, and bioactive compounds they contain (99). They contain various bioactive compounds such as saponins, tannins, flavonoids, isoflavones, lectins, and phytic acid, which help prevent or treat certain diseases such as cardiovascular diseases, diabetes, obesity, overweight, cancer, and digestive system diseases (100).

### 3.1 Bambara groundnut

Bambara groundnut contains higher soluble fibers than other beans, has high protein quality, and also contains various polyphenolic compounds (anthocyanin, catechin, quercetin, kaempferol and its derivatives, isoquercetin, epicatechin, luteolin, rutin, myricetin, chlorogenic acid, p-coumaric acid, ellagic acid, gallic acid, salicylic acid, caffeic acid and its derivatives, t-ferulic acid, quinic acid, and medioresinol). Its main mineral content includes calcium, phosphorus, potassium, and iron (87).

### 3.2 Rice bean seeds

Rice bean seeds offer nutritional benefits, including a high protein content, essential amino acids such as lysine, tryptophan, and methionine, good antioxidant potential, high protein digestibility, and low phytic acid and lectin content. Rice bean seeds contain a variety of saccharides, but are low in gas-producing properties compared to other legumes (101). It also contains several bioactive compounds such as phytate, alpha-galactosides, and trypsin inhibitors, which can act as antioxidants, anti-cancer, and anti-diabetic agents (100).

### 3.3 Millets

Millets are a group of grains rich in protein, calcium, and iron minerals, vitamins, and dietary fibers. They have three to five times the nutritional value of rice and wheat. Due to their slow digestion, millets provide long-lasting energy, making them a good food for diabetics (102). Millets stand out as rich sources of flavonoids such as flavones, flavanols, flavonols, anthocyanins, and proanthocyanidins (103). Due to the bioactive components found in the seed coat (phenolic compounds such as ferulic acid, caffeic acid, vanillic acid, gallic acid, and quercetin), they exhibit antioxidant, anti-carcinogenic, anti-inflammatory, and antiviral properties and have neuroprotective importance against disorders such as cancer, cardiovascular disease, high blood pressure, diabetes, cholesterol, and neurodegenerative diseases (104).

### 3.4 Pseudocereals

Pseudocereals, although not part of the cereal family, stand out among underutilized foods for their properties and uses similar to those of grains. The most representative species are amaranth, quinoa, and buckwheat. High-value proteins and peptides can be obtained from these plants. Other nutritional and bioactive compounds, such as flavonoids, phenolic acids, fatty acids, vitamins, and minerals, can also be obtained (105). Anticancer, antioxidant, and anti-inflammatory properties have been found and hypothesized for peptides derived from pseudocereals. They also contain high amounts of soluble fibers, which help regulate bowel movements and control hypercholesterolemia (high plasma cholesterol levels), hypertension (high blood pressure), and cardiovascular diseases (106). In one study, the highest soluble and bound phenolic contents among three pseudocereals were found in buckwheat flour. The most predominant soluble phenolic acid was dihydroxybenzoic acid, while vanillic acid was the most abundant component in the bound phenolic fractions. All three pseudocereals contain high levels of phosphorus, potassium, and magnesium (107).

### 3.5 Quinoa

FAO adopted the Quinoa Standard CXS 333–2019, which was included in the Codex Alimentarius in 2019, as the quinoa quality standard (108). The main bioactive compounds in quinoa are betaine and polyphenols (kaempferol, rutin, quercetin), and their content increases as quinoa color darkens. Furthermore, quinoa's fatty acid composition is high in unsaturated fatty acids, including omega-3 and omega-6, and has a low omega-6/omega-3 ratio, which may reduce health risks (109). Quinoa is a plant-based protein source containing nine essential amino acids (specifically lysine, methionine, and threonine). These amino acids are generally limited in traditional grains. Quinoa also contains other essential minerals, including magnesium, zinc, iron, calcium, and potassium (110).

### 3.6 Buckwheat

Buckwheat has significant potential as a food ingredient, particularly in the functional and therapeutic food sectors, owing to its high-quality protein, dietary fibers, resistant starch, rutin, vitamins, and minerals. Scientific studies have shown that different parts of buckwheat (seed, flower, sprout, leaf) contain various bioactive compounds, including phenolic compounds, flavonoids (flavonols, flavones, flavanones, isoflavones), triterpenoids, steroids, quinones, glycosides, and many others. Zinc, copper, and potassium absorption from buckwheat is relatively high (111). The most abundant macroelement in buckwheat grains is potassium (112). The bioactive components that enhance the nutraceutical potential of buckwheat are polyphenols (flavonoids and phenolic acids) and flavonoids such as rutin, isoorientin, quercetin, isovitexin, vitexin, and orientin. Among all pseudocereals, rutin is found only in buckwheat and has higher antioxidant, anti-inflammatory, and anticancer properties (76).

### 3.7 Amaranth

Amaranth contains vitamins, such as riboflavin, vitamin C, folic acid, and vitamin E. Amaranth leaves contain antioxidant phytochemicals such as anthocyanins, betalain, β-cyanin, β-xanthin, ascorbic acid, carotenoids, chlorophylls, phenolic acids, and flavonoids. Phenolic compounds such as caffeoylglucaric acid, glucaric isomers, coumaroylglucaric acid, caffeoylquinic acid, feruloylglucaric acid, coumaroylquinic acid, and feruloylquinic acid have been identified in its seeds. These bioactive compounds have anti-ulcer, gastroprotective, anti-colorectal cancer, anti-inflammatory, and anti-oxidant properties, and function as a defense system against various disorders such as atherosclerosis, arthritis, cardiovascular diseases, cataracts, emphysema, retinopathy, and neurodegenerative diseases (113). Amaranth is considered a valuable functional food with the potential to improve overall health and reduce the risk of metabolic disorders. Amaranth is richer in essential amino acids like lysine than traditional grains (114).

### 3.8 Aerial yam

Aerial yam is a unique type of yams known for their ability to produce edible bulbs and tubers that are rich in carbohydrates, low in fat, and have unique functional properties (202). Raw aerial yam poses toxicological challenges due to the presence of significant levels of antinutrient compounds, including alkaloids, saponins, and phenols. Cooking methods enhance the edibility of aerial yams and reduce antinutrient levels to safe levels (115). Boiling was found to be the best method in terms of nutrient content, anti-nutrient factors, and amino acid profile compared to other methods (116).

### 3.9 Taro

Taro is a rich food that contains dietary fibers, protein, starch, soluble sugars, and minerals. Compared to other root crops, taro stands out for its high dietary fiber content. It is also a good source of potassium, magnesium, vitamin C, and vitamin E. Its mineral contents include K, Ca, P, Fe, Mg, Na, and Cu, making it a low-sodium, high-potassium food. The edible root contains a relatively high concentration of flavonoids (28.04 mg) and polyphenols (34.95 mg) (66). It contains bioactive compounds, including flavonoids, alkaloids, and saponins, that exhibit potential antioxidant, antidiabetic, anti-inflammatory, and antimicrobial properties. It is abundant in phenolics and flavonoids such as chlorogenic acid, catechin, and quercetin (117).

### 3.10 Baobab

Baobab is a tree whose fruits and leaves have been traditionally used for their medicinal and nutritional properties. The fruit pulp contains carbohydrates, protein, lipids, fibers, vitamin C, minerals (sodium, potassium, phosphorus, magnesium, iron, zinc, and calcium), and various phytochemicals such as proanthocyanidins, phenolic acids, triterpenoids, flavonol glycosides, and saponins. The phenolic compounds and flavonoids found in the fruit's solvent extracts exhibit exceptional free-radical-scavenging activity, suggesting potential health benefits. They have been linked to various medicinal activities, including anti-inflammatory, antimicrobial, analgesic, antipyretic, anticancer, liver-protective, and antidiarrheal effects. They are also an important source of dietary fibers, which have been linked to promoting satiety, reducing the risk of obesity, and improving intestinal health through their prebiotic properties (67, 118, 119).

### 3.11 Jackfruit

Jackfruit is a tropical fruit rich in carbohydrates, dietary fibers, and protein. It contains vitamins A, B, and C, as well as calcium, iron, magnesium, potassium, and sodium. Phytochemically, jackfruit possesses a valuable profile of bioactive compounds, including polyphenols, flavonoids (quercetin, kaempferol, catechins, and epicatechins), amino acids, volatile acids, carotenoids, and minerals. Preclinical studies indicate that jackfruit exhibits antimicrobial, antioxidant, anti-inflammatory, anti-melanogenic, antidiabetic, immunomodulatory, antiviral, anthelmintic, wound-healing, and antineoplastic properties. Clinical studies indicate that jackfruit leaves have antidiabetic effects in healthy individuals and in individuals with non-insulin-dependent diabetes (120, 121).

### 3.12 Camu-camu

Camu-camu is a tropical fruit with bio-functional properties. It is referred to as a “super fruit” due to its bioactive and antioxidant compounds, such as polyphenols, carotenoids, and vitamin C. It is known as one of nature's most significant sources of ascorbic acid and a potent antioxidant. Its main bioactive compounds are anthocyanins, ascorbic acid, and malic acid. The fruit epicarp contains cyanidin-3-glucoside as the primary anthocyanin pigment, followed by delphinidin-3-glucoside. Anthocyanins are natural pigments that exhibit antidiabetic, anticancer, and anti-inflammatory effects and may prevent cardiovascular and neurodegenerative diseases (122, 123).

### 3.13 Water spinach

Water spinach is a nutrient-rich algae that can be an important part of a healthy diet. It is a good source of amino acids, trace elements (Na, K, Ca, Mg, Zn, Cu, Fe, Mn, and P), and vitamins A, B, C, and E. Furthermore, its bioactive compounds, such as carotenoids, flavonoids, tannins, and steroids, may act as a barrier against free radical-related diseases and degenerative diseases (70, 124).

### 3.14 Moringa

Moringa leaves are regulated as a Novel Food under Regulation (EU) 2015/2283 (125) and included in the Argentine Food Code (CAA), Articles 1192 and 1198 (126).

Moringa is a plant traditionally used to manage diabetes and its complications. Its mechanisms of action include reducing inflammation and improving glucose metabolism and insulin sensitivity. It contains polyphenols, flavonoids, glucosinolates, isothiocyanates, and alkaloids, which have potent anti-inflammatory, anticancer, and antioxidant properties. The most common non-essential amino acid in both the leaves and seeds is glutamine (127, 128). Bioactive compounds such as vanillin, chlorogenic acid, quercetin, kaempferol, gallic acid, and ferulic acid contribute to its therapeutic potential. The leaves have a high iron content, which has been shown to alleviate anemia in pregnant women and children. They have also been linked to a reduced risk of heart problems, diabetes, and cancer (129).

### 3.15 Perilla

Perilla (*P. frutescens*) is widely used in the food, pharmaceutical, and cosmetic industries due to its unique aromatic compounds. Bioactive compounds include essential oils (perillaldehyde, limonene), phenolic acids (rosmarinic acid, caffeic acid), flavonoids (apigenin, luteolin), and triterpenoids. These bioactive compounds exhibit various pharmacological activities, including antioxidant, antimicrobial, anti-inflammatory, antiviral, anticancer, hypoglycemic, and neuroprotective effects. P. frutescens oil, characterized by its high omega-3 fatty acid content (>60%), is considered an important functional food ingredient (130, 131).

### 3.16 Long pepper

Long pepper (*Piper longum*) contains a variety of bioactive compounds, including piperine, pipermundin, piperlongumin, and silvatin. The alkaloid piperine, which gives *Piper longum* its pungency, has numerous effects, including antioxidant, anticancer, anti-inflammatory, antidepressant, immunomodulatory, antihypertensive, analgesic, antiasthmatic, antipyretic, antidiarrheal, anxiolytic, hepatoprotective, antispasmodic, antibacterial, antifungal, antithyroid, antiapoptotic, antimutagenic, antispermatogenic, and antimetastatic. Essential oils from *Piper longum* have been found to contain a variety of phytochemicals, including alkaloids, flavonoids, esters, and steroids. They are reported to be antimicrobial, anti-inflammatory, analgesic, antioxidant, anticancer, antiparasitic, anthelmintic, antiarthritic, antiulcer, antiasthmatic, cardioprotective, mosquito larvicidal, neuropharmacological, antihyperglycaemic, hepatoprotective, antihyperlipidemic, antiangiogenic, immunomodulatory, and anti-snake venom agents (98, 132, 133).

As in the NUS examples examined in this section, biochemical constituents determine pharmacological properties. These bioactive compounds can also be used to design functional food products (99). Species, environmental, and developmental factors appear to influence the biochemical profile of plants. To address these limitations, studies on plant standardization are needed.

## 4 Current methods of phytochemical extraction from NUS

In recent years, the increasing demand for functional foods, the growing significance of natural antioxidant sources, and global efforts to conserve biological diversity have intensified interest in the phytochemical potential of NUS. One of the most critical stages in unlocking this potential is the effective extraction of bioactive compounds. Since extraction efficiency directly influences the quantity, purity, and bioavailability of the obtained compounds, it has become one of the key factors determining the industrial value of NUS (18).

In addition to conventional solvent extraction techniques, more efficient, selective, and environmentally friendly methods have emerged in recent years. Ultrasound-assisted extraction, microwave-assisted extraction, supercritical fluid extraction, and green solvent–based approaches have attracted attention for their ability to shorten processing time, reduce energy requirements, and minimize solvent consumption. However, the morphological diversity and distinct phytochemical profiles of NUS necessitate the evaluation of the most suitable extraction technique for each species individually (134).

**Figure 4:**
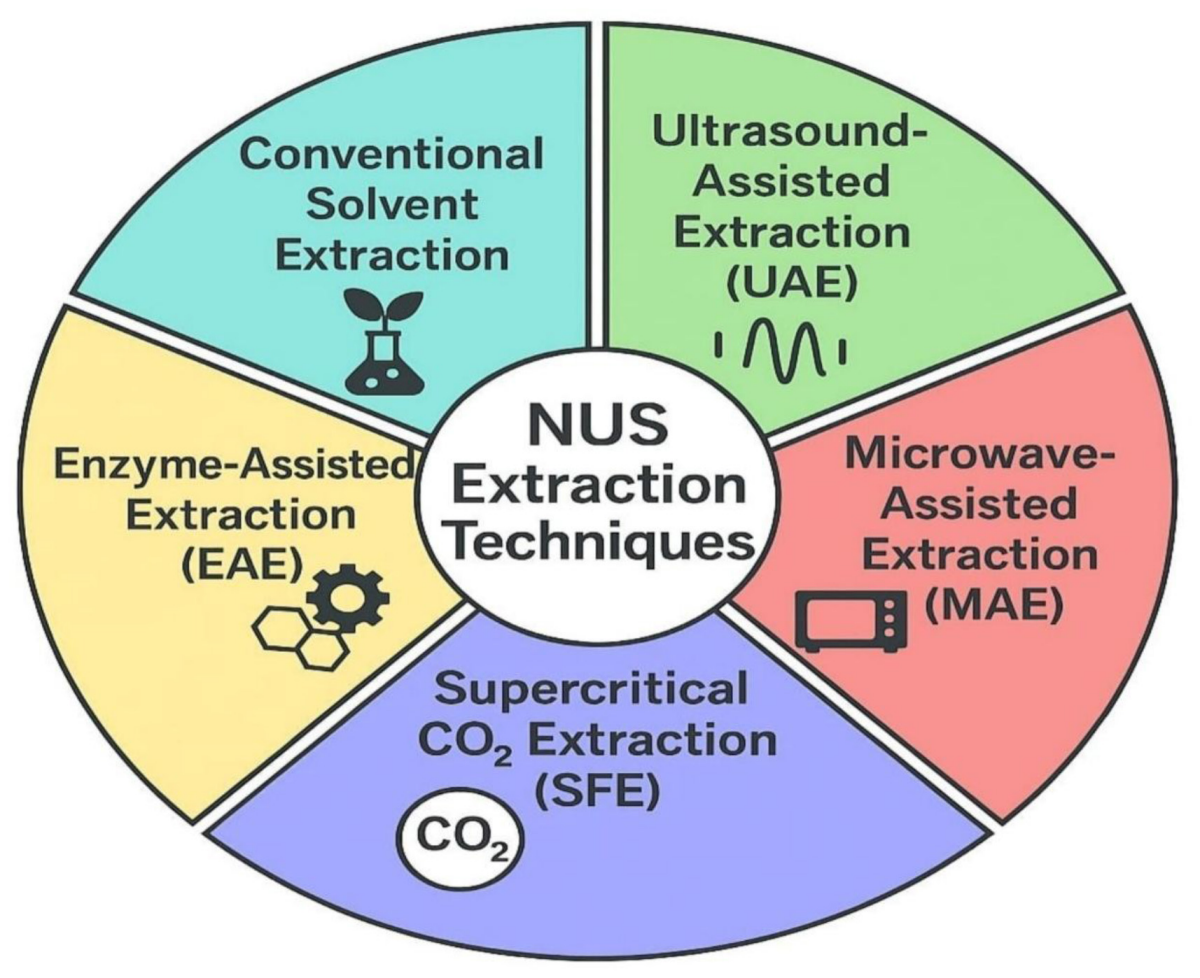
Primary extraction methods employed for the recovery of phytochemicals from NUS.

In a period marked by rapidly increasing demand for natural compounds, the development of extraction methods for obtaining phytochemicals from NUS supports sustainable production goals and enhances the economic value of these species. This section examines up-to-date extraction techniques applied to NUS and evaluates the current state of the field within the framework of technological advancements and sustainability-oriented approaches. The extraction methods used to obtain phytochemicals from NUS are illustrated in Figure 4.

### 4.1 Conventional solvent extraction

Solvent extraction is an extraction technique in which a component is removed from a solid matrix using a liquid solvent. Parameters such as solvent type and concentration, sample particle size, temperature, duration, and product-to-solvent ratio, as well as the extraction technique employed, play a decisive role in phytochemical extraction.

Different phytochemical groups differ markedly in polarity and stability, and therefore require different extraction approaches. Polar phenolic compounds (including flavonoids) are generally more amenable to polar solvent-based techniques (e.g., solvent extraction, maceration, UAE, MUAE). Less polar or non-polar compounds, such as many terpenoids and essential oils, are more efficiently recovered using distillation or supercritical fluid extraction (SCFE).

Conventional solvent extraction remains one of the most widely used methods for obtaining phenolic compounds in studies conducted on NUS. Research performed on plants such as amaranth leaves, *Moringa oleifera*, Bambara groundnut, and various millet species has demonstrated that phenolic compounds with high solubility can be isolated with substantial yields using this technique (135).

Traditional methods for extracting phytochemicals from plants, such as maceration and Soxhlet extraction, are more frequently used in analytical and laboratory-scale studies. Pressing extraction, by contrast, is used in specific industrial applications. Soxhlet extraction is used for comparison purposes and allows the determination of the total amount of plant material extractable with a given solvent. Maceration is used mainly for analytical purposes and has specific industrial applications. It is carried out at moderate temperatures or at room temperature. Maceration and Soxhlet extraction are characterized by high energy consumption, excessive solvent use, and long extraction times, while mechanical pressing generally has disadvantages such as low extract yield (136).

Therefore, recent studies on NUS have begun to utilize green extraction methods with higher industrial-scale applicability, such as ultrasound, microwave, and supercritical fluid extraction, to use smaller amounts of solvent, increase extraction efficiency, and shorten processing time (135).

### 4.2 Green extraction method

With the United Nations (UN) prioritizing a more sustainable future, the significance of greener extraction methods has become increasingly evident. Particularly in the extraction of phytochemical compounds, approaches that prevent environmental harm have come to the forefront. These techniques are being increasingly preferred for phytochemical recovery as they offer advantages such as higher mass transfer efficiency, shorter processing time, reduced solvent and energy consumption, and improved preservation of heat-sensitive compounds (137). Recent studies on NUS have demonstrated that green extraction methods not only shorten processing time but also substantially enhance extraction efficiency by preventing the loss of bioactive components. Literature reviews on NUS indicate that among the most widely investigated extraction techniques are supercritical CO_2_ extraction, ultrasound- and microwave-assisted processes, and enzyme-assisted extraction, depending on the species, the plant part, and the nature of the target phytochemicals.

#### 4.2.1 Supercritical CO_2_ extraction (SC-CO_2_)

Supercritical fluids are defined as substances that exist above their critical temperature and pressure, where no distinct liquid–gas phase boundary is observed (138). Supercritical carbon dioxide (SC-CO_2_) is widely preferred for the extraction of food-based bioactive compounds due to its non-toxicity, non-flammability, economic feasibility, and environmentally safe solvent characteristics (139). Compared with conventional solvent extraction, supercritical fluid extraction (SFE) with SC-CO_2_ has been reported to reduce solvent use by approximately 80%−90% (140). Owing to these properties, SC-CO_2_-assisted extraction has become increasingly prevalent in NUS-focused research. Studies conducted on baobab, perilla seed, *Moringa oleifera* leaves, jackfruit seed, and various oil-rich NUS species have demonstrated that SC-CO_2_ yields particularly high extraction efficiency for lipophilic compounds, essential oils, and carotenoids. Selected studies in the literature reporting the use and outcomes of supercritical extraction on NUS are summarized in Table 5.

**Table 5:** Supercritical extraction parameters and results applied to phytochemicals in some NUS species.

**Plant**	**Method**	**Method parameters**	**Phytochemicals**	**References**
*Moringa oleifera* leaves	SFE-CO_2_ using different co-solvent mixtures: CO_2_ + water/ethanol. Pressure, temperature, and time parameters optimized.	Extraction conditions: 100 bar, 80 °C, 120 min	Its effect on total flavonoid extraction was determined, and the highest total flavonoid content was 11.66 mg TFC/g.	(141)
*Moringa oleifera* leaves	Experimental variables, including temperature (35–80 °C), pressure (15–25 MPa), and ethanol-to-raw material mass ratio, were investigated.	Central point extraction conditions were determined as 60 °C and 20 MPa	The phenolic compound profile revealed that gallic acid, vanillic acid, and p-coumaric acid were identified as phenolic acids in the extracts, while catechin was identified among the flavonoid compounds.	(142)
*Perilla frutescens* leaves	Classical SC-CO_2_ and ultrasound-assisted supercritical CO_2_ (USCCO_2_) methods were used.	USCCO_2_ achieved 1.12–1.92 times shorter extraction duration and 1.90–285.71 times lower solvent consumption compared to classical SC-CO_2_ and thermal reflux	It was used to extract caffeic acid and rosmarinic acid. The extraction yields of rosmarinic acid and caffeic acid obtained by the supercritical fluid method were determined as 26.47 mg/g dry plant and 1.96 mg/g dry plant, respectively.	(143)

However, when supercritical fluid extraction is applied with systems containing organic solvents such as ethanol, some studies have determined that the extraction yield and total flavonoid content are lower compared to supercritical methods without auxiliary solvents (141). These findings indicate that method parameters and solvent systems should be carefully optimized to achieve high yields of phytochemicals in supercritical fluid extraction.

In conclusion, studies conducted specifically on NUS clearly demonstrate the effectiveness of SFE, as evidenced by the high yields of total phenolics and flavonoids achieved under optimized conditions. Therefore, SFE stands out not only as a technique that enhances extraction efficiency but also as an innovative approach that supports sustainable bioavailability by converting NUS resources into high-value bioactive compounds.

#### 4.2.2 Ultrasound-assisted extraction

Ultrasound-assisted extraction (UAE) is recognized as an environmentally friendly and highly efficient extraction technology (144). Ultrasound waves are mechanical vibrations characterized by pressure variations, particle displacement, and velocity differences in an elastic medium; when their frequency falls within the ultrasonic range, these vibrations are termed ultrasonic waves. The fundamental principle of the UAE relies on cavitation, which is induced by ultrasonic waves applied to the liquid phase. The resulting physical effects accelerate the rupture of plant cell walls, thereby facilitating the transfer of phytochemical constituents into the solvent (145).

Ultrasound-assisted extraction offers several advantages over conventional extraction techniques, including operation at lower temperatures, reduced energy consumption, shortened extraction time, and preservation of extract quality (146). Through cavitation-induced cell disruption, which enhances the diffusion of phytochemicals into the solvent, UAE enables rapid and efficient recovery of phenolic compounds from NUS species. Studies conducted on species such as amaranth, quinoa, and water spinach have reported substantial increases in polyphenol content and antioxidant capacity following ultrasonic treatment.

#### 4.2.3 Microwave-assisted extraction

Microwave-assisted extraction (MAE) has also emerged as an innovative extraction technology, attracting increasing attention for the recovery of phytochemicals. This method relies on the direct, rapid heating of plant material using microwave energy. The instantaneous rise in intracellular temperature leads to the rupture of cell walls, enabling the accelerated release of bioactive compounds into the solvent phase (147). Moreover, its controlled thermal effect minimizes the degradation of heat-sensitive phytochemicals. Recent studies on NUS species further demonstrate that MAE enhances the extraction yields of phenolic and flavonoid compounds, enabling the effective valorization of the bioactive potential of these crops. Selected studies evaluating the effects of microwave- and ultrasound-assisted extraction on phytochemical yield in NUS are presented in Table 6.

**Table 6:** Selected studies applying microwave- and ultrasound-assisted extraction for the recovery of phytochemical compounds from NUS.

**Plant**	**Method**	**Method parameters**	**Phytochemicals**	**References**
Amaranth **(***Amaranthus hypochondriacus* var.)	Ultrasound-assisted extraction	For extraction optimization, temperature (25.86–54.14 °C) and ultrasonic power density (UPD; 76.01–273.99 mW/mL) were evaluated as variables.	Total betalains (BT), betacyanins (BC), betaxanthins (BX), and total polyphenols (TP) were evaluated. At optimum conditions, BC, BX, and BT were 107.42, 42.58, and 149.41 mg/100 g d.m., respectively, and TP reached 1296.49 mg GAE/100 g d.m.	(148)
Jackfruit (*Artocarpus heterophyllus* Lam*.)* pulp	Various extraction techniques, including classical extraction, US, MAE, and US + MAE (UMAE).	UAE was applied at 250 W for 15 min; MAE at 550 W for 165 s; and UMAE at 250 W ultrasound power and 550 W microwave power for 165 s.	Total phenolic content and phenolic profile were evaluated. The highest TPC (2.40 mg GAE/g) was obtained by MAE using 60% ethanol; 43 phenolic compounds were identified.	(149)
Baobab (*Adansonia digitata*)	UAE optimized using response surface methodology (RSM).	As part of the extraction optimization, the following parameters were selected: extraction time (10–20 min), temperature (40–60 °C), and ultrasonic amplitude (30%−50%).	Total phenolic content, total flavonoid content, and phenolic profile were examined. A total of 10 compounds were identified within the phenolic profile, with D-(+)-catechin being the highest concentration (87.18 ± 14.57 μg/g DM).	(150)
Bambara groundnut	Microwave-assisted extraction	Microwave-assisted extraction was performed for 5 and 8 min, with microwave power ranging from 900 to 1,200 W.	Flavonoids (e.g., catechin, quercetin) and phenolic acids (protocatechuic and gallic acids) were investigated in Bambara seeds. Microwave treatment caused increases and decreases in individual flavonoids, including catechin, quercetin-3-O-glucoside, and hesperidin.	(151)

Overall, the literature indicates that both MAE and UAE markedly enhance the recovery of phytochemical compounds from NUS species. These technologies not only increase total phenolic/flavonoid content and antioxidant activity but also significantly reduce extraction time. Such outcomes clearly demonstrate that MAE and UAE are highly efficient and preferable methodologies that substantially improve phytochemical extraction performance, ensuring the effective and sustainable recovery of bioactive compounds from NUS.

#### 4.2.4 Enzymatic extraction

Enzyme-assisted extraction (EAE) is an innovative and environmentally friendly extraction technique that enables the efficient recovery of phytochemicals from plants and agricultural food by-products under mild processing conditions (152).

Enzyme-assisted extraction significantly enhances extraction efficiency by promoting the selective release of intracellular bioactive compounds through the controlled hydrolysis of polysaccharides and other cell wall–bound constituents (153).

Wild fruits such as baobab (*Adansonia digitata*) possess a rich phytochemical profile characterized by high levels of ascorbic acid and flavonoids. Enzyme-assisted extraction enables the liberation of anthocyanins and phenolic acids bound within the peel and pulp matrices, resulting in higher extraction yields than conventional solvent-based approaches. The phenolic-rich extracts obtained through this method hold potential for use in functional beverages and as natural preservative additives (154).

Bambara groundnut (*Vigna subterranea*), *Macrotyloma unilorum*, and jackfruit seeds are valuable botanical raw materials due to their protein fractions and diverse secondary metabolites. Enzyme-assisted extraction using proteases in these species has been shown not only to accelerate the release of bioactive peptides but also to enhance protein digestibility and nutritional quality (155). These peptides possess antioxidant and antimicrobial activities, underscoring their potential to enhance functional food formulations and other food applications.

Studies on NUS in the literature demonstrate that EAE targets cell wall polysaccharides, releasing bound phenolic fractions and thereby markedly increasing total phenolic content (TPC), total flavonoid content (TFC), and antioxidant activity. Therefore, EAE has emerged as a promising technique for the high-yield and low-temperature extraction of NUS-derived phytochemicals.

### 4.3 Challenges in extracting phytochemicals from NUS

Although significant progress has been made in improving phytochemical recovery from NUS species, the extraction process is still constrained by several limitations. One of the primary challenges arises from the heterogeneous nature of plant raw materials. Many NUS species exhibit structural features, such as high fiber content, variable moisture levels, and thick cell walls, that hinder solvent infiltration and compound release from the matrix. Furthermore, the phytochemical composition of NUS varies substantially across species; therefore, an extraction method that yields optimal results for one species may not yield comparable results for another. There is a need to match solvent polarity with the target phytochemical class of NUS. Polar solvents favor the recovery of phenolic compounds, whereas less-polar or non-polar systems are more suitable for isoprenoids; alkaloids often require pH- or polarity-adjusted conditions. Therefore, a range of solvents with varying polarities will be required to recover a wide variety of phytochemicals.

Another major challenge lies in the limited characterization of the chemical profiles of many NUS species. The scarcity of comprehensive compositional data for certain species complicates the rational optimization of extraction conditions. In addition, pre-processing variables such as post-harvest losses, storage duration, grinding, and the use of fresh vs. dried material significantly influence extraction performance.

From a technological standpoint, high equipment costs, long processing times, and elevated energy demands remain critical barriers to the adoption of advanced extraction methods, particularly in developing regions with limited research infrastructure. Consequently, for environmentally friendly extraction strategies to be implemented at larger scales, improvements are required in cost-effectiveness, practical applicability, and technical accessibility.

## 5 Hydrogen as a novel and sustainable extraction aid

### 5.1 Properties and advantages of molecular hydrogen (H_2_)

Molecular hydrogen (H_2_) is nature's simplest and smallest element. Molecular hydrogen (H_2_) is found in very low levels (0.53 ppm) in the world. It is a colorless, tasteless, and inert gas. In other words, it is a gas that does not spontaneously initiate reactions in its environment. This property gives hydrogen many advantages. For example, it forms a safe structure in biological systems because it does not react with its environment. It does not produce toxic by-products. It allows the reaction to occur in the environment in which it is present. H_2_ is non-polar, which enables it to penetrate tissues readily (156). Additionally, hydrogen has the potential to reduce environmental pollution. H_2_, the simplest and most common molecule, is valuable for its properties as a selective antioxidant and anti-inflammatory agent. Given these properties, hydrogen has recently emerged as a green extraction method that can serve as an alternative to traditional solvents for phytochemical extraction and the utilization of agricultural food waste (157, 158).

### 5.2 Mechanisms of action in biological systems

Today, H_2_ has come to the fore due to its unique bioactive properties. In this context, it particularly reduces oxidative stress by neutralizing reactive oxygen and nitrogen species without disrupting cellular functions. Indeed, Ohsawa et al. (159) reported that H_2_ reduces reactive oxygen species (ROS) that damage brain cells. Additionally, the beneficial effects of H_2_ have been documented in numerous clinical and animal studies, as well as in studies on food quality preservation. The biological activity of H_2_ at low concentrations remains incompletely understood, and its mechanism has been only partially addressed. A review of the studies conducted indicates that most reports have focused on antioxidant, anti-inflammatory, and anti-apoptotic effects. Hydrogen has been found to suppress signaling pathways in allergic and inflammatory responses in the system where it is present (160). The antioxidant effect of hydrogen can be explained, in particular, by the direct elimination of hydroxyl radicals and peroxynitrite. Thus, in the presence of H_2_ in the biological system, cells can be protected. In addition, H_2_ can be used on its own in new, sustainable green extraction processes and combined with various extraction techniques (158).

Understanding the mechanisms underlying the use of H_2_ in foods (for phytochemical extraction) and in plants is quite important (161). Reactive oxygen species (ROS) and reactive nitrogen species (RNS) play critical roles in plant responses to stress. These structures disrupt sensitive redox homeostasis and cause cellular damage within plant cells. They speed up the aging process in plants. Plants produce endogenous H_2_ under stress. H_2_ activates antioxidant enzymes, such as catalase and superoxide dismutase. Furthermore, H_2_ minimizes the harmful effects of heavy metals (e.g., cadmium) by regulating calcium signals and activating enzymes (RbohD) (162–164). H_2_ enables the plant to absorb Sulfur. This is particularly important when the plant is under stress. H_2_ activates the genes responsible for Sulfur metabolism in the plant (ATP sulphurylases, etc.) (165). H_2_ also affects plant flavonoid metabolism. It performs this function primarily by activating genes and enzymes responsible for anthocyanin metabolism. It also regulates calcium signals under stress (166, 167). H_2_ also plays an important role in photosynthesis. It protects the plant against temperature stress factors, increasing chlorophyll levels. Again, under the same conditions, it plays a role in carbon and nitrogen metabolism, stimulating the relevant enzymes. This increases the nitrogen and sugar content within the cell (166, 167). H_2_ also affects plant hormones. It contributes to plant growth and rooting, particularly by affecting certain hormones (ABA, ethylene) (168, 169). The oxidation of phenolic compounds generates quinone-derived reactive species. Quinones also damage products by binding to reducing agents in the environment. H_2_ present in the environment slows down the oxidation of phenolics, reducing quinone formation. It also contributes to the process by binding hydroxyl radicals in the environment (170, 171). H_2_ exhibits selective antioxidant properties against the strongest radicals (• OH and ONOO^−^). H_2_ plays a role in the electron transfer system. This enables it to protect phenolics and antioxidants from oxidative reactions (172).

### 5.3 Current applications of hydrogen in plant sciences

**Figure 5:**
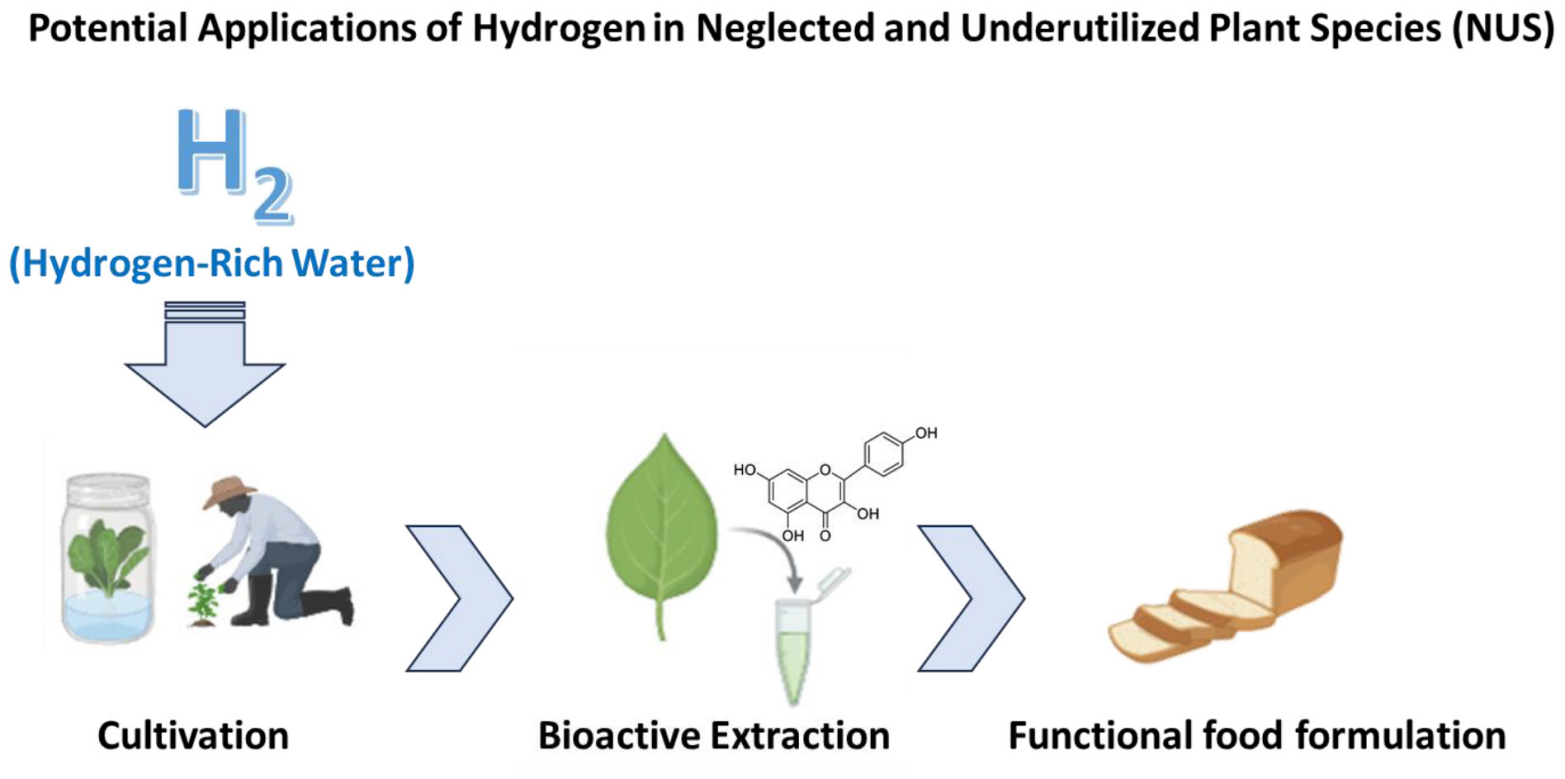
Potential applications of hydrogen in neglected and underutilized plant species (NUS).

The use of hydrogen in the plant field has been reported in the literature. It is known that hydrogen can be applied in various forms (hydrogen gas, hydrogen-rich water, hydrogen-rich nano-bubbled water, magnesium hydride, etc.) (Table 7). Figure 5 shows the potential applications of hydrogen in NUS.

**Table 7:** Use of hydrogen applications in some plants.

**Use type**	**Materials**	**Functions of H_2_**	**References**
HRW	Soak seeds	It can stimulate the growth of shoots and roots	(168)
HRW	Kiwifruit	Delayed ripening and senescence	(173)
H_2_	Kiwifruit	Ethylene gas control	(174)
HRW	Okras	Delayed ripening and senescence	(175)
HRW/H_2_	Tomato	Minimizes nitrite accumulation	(176)
HRW	Banana	Delayed ripening and senescence	(177)
HRW	Chinese water chestnut	Reduction in tissue yellowing, reduction in oxidative stress	(178)
HNW	Carnation (*Dianthus caryophyllus*)	Slowed oxidative stress, prolonged flower life	(179)
HRW	Lemon peel	Increased extraction efficiency Increased phenolic and flavonoid content	(180)
H_2_	Green tea leaves	Increased extraction efficiency of phenolics	(181)

#### 5.3.1 Hydrogen gas

It is widely used in food and plants and is highly flammable. As hydrogen gas is flammable, it poses a significant risk (182). There are application difficulties due to factors such as production, cost, and safety. Additionally, there is the H_2_ rapid-escape issue.

#### 5.3.2 Hydrogen-rich water (HRW)

Hydrogen is preferred primarily to increase the release time of H_2_, improve its retention time, and enhance its biological effect. Preparing HRW is an easy method. Hydrogen dissolves in regular water until it reaches saturation (approx. 1.6 mg/L).

#### 5.3.3 Hydrogen-rich nano-bubbled water (HNW)

Nano-bubbles have diameters less than 500 nm, high internal pressure, and large surface area. This method will improve H_2_ dissolution and extend processing times (183).

#### 5.3.4 Production of H_2_ gas from magnesium (Mg)

H_2_ gas is produced as a result of the reaction of magnesium (Mg) with water. This is another simple process to prepare HRW.

### 5.4 Rationale for hydrogen-assisted extraction (H2Ext)

In recent years, H2Ext has emerged as a low-cost, non-toxic, and safe green extraction method (184). Molecular hydrogen (H_2_) is a reducing gas with over 30 well-known biological properties (156). H_2_ gas is lipophilic and small in size. This property enables passive diffusion into cell membranes and tissues, where it readily penetrates. It has been reported that H_2_ can be added to different solvents (21). It imparts a reducing property that may help maintain redox homeostasis within the cell during the extraction process. This protects the phenolics and antioxidants in food or plants from oxidative reactions.

Hydrogen can be used to enrich extraction solvents (e.g., water, ethanol, methanol, hexane) in various plant species. Elnasankasim et al. (200) used hydrogen extraction to recover phenolic compounds from tea waste. For phenolic extraction, they used hydrogen-rich water (HRW), magnesium water (Mg water), ethanol/Mg water (1:1), and ethanol/pure water (1:1). The phenolic compound catechin was detected with HRW. Additionally, although chlorogenic acid was not detected in pure water extracts of tea waste, it was detected in HRW and Mg water samples at concentrations of 904.32 and 550.47 μg/g extract, respectively.

On the other hand, hydrogen can be used alongside CO_2_ at a safe level (less than 4%, v/v) in the supercritical CO_2_ extraction process known as hydrogen-incorporated CO_2_ extraction (Sp-CO_2_-H_2_). In a modeling study using Sp-CO_2_-H_2_ extraction to recover phytochemicals from grape peels, significant differences were observed in the phenolic compounds t-ferulic acid and catechin with the extraction method (199). No studies have been conducted on NUS using this novel method.

It has been reported that the use of hydrogen-rich water alone or in combination with ethanol improves the extraction of various phytochemicals. The phytochemical extraction rate with HRW increased by 0.5–4 times, with the most significant improvement observed in anthocyanins. Significant increases were observed in the amounts of certain flavonoids and non-flavonoid compounds, particularly rutin and epicatechin (158). Yurt (24) investigated the effect of hydrogen-enriched solvents on propolis. HRW was the best extraction solvent for gallic acid, chlorogenic acid, caffeic acid, p-coumaric acid, trans-ferulic acid, rosmarinic acid, catechin, and epicatechin. HRM yielded the best results for quercetin in propolis samples. When hydrogen was added to methanol, ethanol, and water, an increase in extraction yield (24.3%, 20.6%, and 18.6%, respectively) was observed. Alwazeer et al. (180) reported that the addition of H_2_ to solvents resulted in a significant increase in the total anthocyanin content of red beetroot extract. Increases of 92.62%, 199.5%, and 257.41% were observed for water, ethanol, and methanol, respectively. Hydrogen-rich solvents improved the extraction of certain hydroxybenzoic acids, including gallic and syringic acids. However, they reduced the extraction of others, such as vanillic acid, 3,4-dihydroxybenzoic acid, and 4-hydroxybenzoic acid. Additionally, the extraction of some flavan-3-ols, including catechin and procyanidin B2, has been increased by hydrogen-rich solvents. Epicatechin, by contrast, has decreased (180).

The hydrogen-rich solvent extraction reported in the literature has been applied only to endemic plants (cowslip and primrose). Cowslip (*Primula veris* L.) is a wild plant that grows abundantly in various regions, including Turkey. It contains various bioactive compounds, including triterpenes, saponins, flavonoids, phenolic acids, and phenolic glycosides. It is used in the production of tea and medicine. It is a wild plant that grows in tropical climates. Additionally, the leaves of this plant are used in gastronomy (185). Engin et al. (186) examined the hydrogen extraction (H2Ext) method for the recovery of phytochemicals from cowslip flowers. The authors tested several hydrogen-rich solvents (water, methanol, ethanol). They concluded that hydrogen-rich methanolic extracts yielded higher pigment levels, flavonoid and phenolic contents, antioxidant activities, and pigments (chlorophyll, β-carotene, and lycopene).

Salmanca (*Chenopodium album* L.) is a nutritious and edible wild plant grown in many regions, such as Turkey (187). Salmanca is rich in bioactive compounds, such as phenolic acids and flavonoids. It also has a balanced amino acid profile and high-quality protein, making it a suitable nutritional choice. The leaves of Salmanca contain high levels of coumaric acid, while the fruit contains high levels of gallic acid. Furthermore, the literature reports that Salmanca exhibits anti-inflammatory (188), anti-rheumatic, anti-ulcer, anti-diabetic, and anti-hyperlipidaemic activities (189). Özdilek (190) investigated the use of hydrogen extraction (H2Ext) using various solvents (water, ethanol, and methanol) on the extraction of phenolic compounds (TPC), flavonoids (TFC), antioxidants, pigments (chlorophyll), sugars, and organic acids from Salmanca. The author concluded that the highest yield was obtained for hydrogen-rich methanol extracts.

As a result, the increase in phytochemicals observed in samples extracted with H_2_ opens the door to different explanations (24). During extraction, hydrogen can protect phenolic compounds from both enzymatic and non-enzymatic oxidative reactions and facilitate the release of phenolics bound to the cell wall.

## 6 Limitations, knowledge gaps, and future research directions

### 6.1 Technical challenges in hydrogen use

H2Ext is now explicitly presented as a complementary, matrix-assisted green extraction approach, whose applicability is primarily limited to certain polar or moderately polar phytochemicals when combined with appropriate solvent systems, rather than as a universal extraction method for all phytochemical classes.

Despite the potential of hydrogen technologies in the food industry, several technical limitations impede their widespread adoption. Firstly, concerns about the flammability and explosiveness of hydrogen have increased the demand for equipment and process safety. A hydrogen-air mixture with an H_2_ concentration of approximately 4%−75% by volume is capable of exploding in the presence of an ignition source (191). Therefore, all operations involving hydrogen (generation, bubbling into a liquid, storage) require strict control, including the use of explosion-proof equipment, ventilation, and the exclusion of open flames. These safety measures complicate production and increase its cost. Secondly, there are difficulties with the long-term storage of hydrogen-rich products. Molecular hydrogen diffuses readily through most materials, making it challenging to keep it dissolved in liquid for long periods. The need for specialized multilayer packaging (e.g., based on aluminum foil) somewhat constrains product form factors and increases costs. Furthermore, the H_2_ concentration in the product decreases over time, especially at higher temperatures or when containment is breached. Therefore, ensuring a stable hydrogen concentration from production to consumption remains a challenging task (192).

Another technical challenge is dosing and monitoring H_2_ content. Currently, there are no generally accepted standards for the optimal hydrogen level in specific products to achieve the desired effect. Hydrogen concentrations, expressed in ppm (parts per million) or mol/L, can vary significantly across commercial hydrogen water samples. This lack of standardization hinders the comparison of research results and consumer confidence (193). Equally important, large-scale methods for directly generating hydrogen in food processes remain underdeveloped. Laboratory gas sparging systems do not always scale linearly to industrial production levels, especially for uniformly saturating large volumes of liquid. Technical challenges also include integrating hydrogen technologies with existing production lines: equipment must be adapted, personnel must be trained in the safe handling of H_2_, and gas monitoring systems must be implemented. Addressing these issues will require the interdisciplinary efforts of engineers, chemists, and food technologists (194).

### 6.2 Need for mechanistic and kinetic studies

Although the overall beneficial effects of hydrogen on phytochemical extraction and human health have been demonstrated in numerous studies, the mechanisms underlying these effects remain unclear. In the context of plant extraction, the manner in which H_2_ interacts with the cellular structures of plant materials is unknown. It is possible that hydrogen acts as a reducing agent, preventing the oxidation of target compounds, or that it ruptures cell walls by forming microbubbles, facilitating the leaching of phytochemicals. However, direct studies elucidating the kinetics of H_2_ extraction processes remain lacking. For example, it is unclear how the hydrogen flow rate, pressure, or bubble size affects the extraction efficiency of different compound classes. Optimizing hydrogen extraction technologies requires in-depth research, including the variation of process parameters and the mathematical modeling of mass transfer in the presence of dissolved gas (195).

Questions also remain in the biomedical context. Although H_2_ is known to act as an antioxidant and to modulate signaling pathways, the specific molecular targets of hydrogen in the cell remain to be determined. Activation of Nrf2-dependent genes, effects on mitochondrial function, and modulation of inflammatory cascades (e.g., NF-κB) are hypothesized but require confirmation. The kinetics of hydrogen distribution in the body are also not fully understood: how quickly after consumption, hydrogen water delivers H_2_ to various organs, the half-life of H_2_, and how long elevated hydrogen levels in tissues are maintained. Answers to these questions would allow for rational planning of hydrogen product consumption regimens to achieve maximum benefit (e.g., frequency of intake, dosage) (196).

There is also a gap in understanding the long-term effects of hydrogen. Most studies evaluate indicators immediately after a short course of H_2_ intake (several days or weeks). It is unknown whether multi-month or multi-year consumption of hydrogen water leads to sustained changes in metabolism or disease prevention, or, conversely, to adaptive responses that reduce effectiveness (e.g., increased prooxidant signaling in response to constant external antioxidant exposure). Answering these questions will require both long-term observational studies in humans and experiments in cellular and model organisms to elucidate the fundamental mechanisms of H_2_ action. Ultimately, in-depth mechanistic and kinetic studies will provide the scientific basis necessary for the justified use of hydrogen in food science and medicine.

### 6.3 Regulatory and safety considerations

Another set of restrictions concerns regulatory and safety issues associated with the introduction of hydrogen-containing products to the market. On the one hand, molecular hydrogen is already recognized as a safe component: for example, the US Food and Drug Administration (FDA) has classified H_2_ as generally recognized as safe (GRAS) for addition to beverages at concentrations up to ~2% by volume (197). This means that, from a toxicological standpoint, consuming hydrogen in the specified amounts poses no harm. However, GRAS status does not resolve all issues. Many countries still lack clear regulations regarding hydrogen water or similar products. There are no specific labeling standards (e.g., the H_2_ concentration that must be guaranteed and how it should be indicated on the label), and there are no categories for registering such products (they are neither vitamins, drugs, nor regular dietary supplements).

Regulatory uncertainty complicates the market launch of hydrogen products. Manufacturers face the possibility that any claimed benefits of hydrogen could be considered undeclared medical claims requiring proof. Health authorities rightly require extensive clinical trials before officially marketing hydrogen water, for example, as a means of reducing inflammation or protecting against disease. Until such large-scale evidence is available, hydrogen drinks can only be promoted as general health products without specific therapeutic claims.

As for process safety, some issues require regulatory consideration. Industrial use of hydrogen in the production of food ingredients must comply with explosion safety and sanitary regulations. New protocols and certifications may be required for facilities using H_2_ (similar to those required for food production using ammonia or carbon dioxide). The lack of precedent for hydrogen use in the food industry (except for the hydrogenation of oils) means regulators may approach these innovations with particular scrutiny. In the long term, overcoming these limitations requires: (1) developing standards—for example, establishing recommended hydrogen levels in water, measurement methods, and shelf-life guarantees for H_2_ content; (2) accumulating clinical data to confirm the claimed effects—this will allow for the legal codification of acceptable health claims; (3) collaborating with regulatory authorities to develop an adequate classification of hydrogen-containing products (possibly introducing a new category of functional beverages). Addressing these challenges is essential for ensuring that safe and effective hydrogen-fortified products can find a place in the market and gain consumer trust.

## 7 Conclusion

NUSs are increasingly discussed not because they are “new,” but because modern food systems have tended to ignore them. Many of these plants are nutritionally rich, locally adapted, and resilient under harsh growing conditions—traits that matter as climate pressures intensify and food security becomes more fragile. NUS also offers phytochemical diversity that is often absent from mainstream crops, making it a promising resource for micronutrient improvement and diet diversification, particularly in regions affected by “hidden hunger.”

However, NUS will not move from potential to impact without improved processing and value chain development. This is where green technologies—hydrogen-enabled extraction included—could help bridge the gap. Cleaner extraction approaches can produce stable, high-value ingredients from NUS while reducing environmental burden and preserving bioactivity. In the long run, pairing NUS with sustainable processing methods supports a broader strategy: diversify what we grow, improve what we extract, and produce food ingredients that are both health-relevant and environmentally responsible.

Molecular hydrogen (H_2_) has a small but interesting profile for food and health applications. It is colorless and odorless and is generally considered safe at moderate levels. What makes it different from many additives is that it can act as a selective antioxidant, targeting highly reactive radicals (such as the hydroxyl radical) without broadly disrupting redox processes that are part of normal cellular signaling. Because hydrogen is the smallest molecule and electrically neutral, it diffuses quickly and can move across biological membranes with ease. Alongside direct radical scavenging, a growing body of work suggests that H_2_ may influence cellular defense systems and inflammatory responses, which helps explain why it has attracted attention beyond introductory chemistry.

From a processing perspective, hydrogen becomes most relevant when extraction conditions damage the very compounds we seek to recover. Many conventional extraction methods rely on high temperatures, long processing times, or solvent systems that can promote oxidation and breakdown of sensitive phytochemicals. Introducing hydrogen into the extraction environment may reduce oxidative degradation and help preserve labile molecules during processing. Several reports indicate that hydrogen-enriched solvents can improve the recovery of polyphenols, flavonoids, and other antioxidant compounds, suggesting that hydrogen can play a practical “protective” role during extraction. However, hydrogen-assisted extraction can be considered as complementary rather than substitutive to solvent selection. Specifically, H_2_ is a green auxiliary factor that may influence mass transfer, redox stability, or matrix disruption, whereas the primary extraction selectivity remains governed by solvent polarity and plant-matrix characteristics.

The appeal is not only about yield. When oxidation is reduced, extracts typically contain fewer degradation products and may remain stable for longer, which matters for both functionality and product consistency. From a sustainability standpoint, hydrogen-assisted approaches also align with the principles of green chemistry: they may enable milder conditions, reduced solvent use, and lower energy input, depending on the system design. If these methods can be standardized and scaled, hydrogen could become a valuable addition to the toolkit of sustainable phytochemical extraction—primarily for delicate bioactive targets.

Hydrogen-fortified products—particularly beverages—are already on the market, indicating consumer curiosity and commercial momentum. Looking ahead, the concept could expand into more familiar formats (tea, coffee, juices, recovery drinks), but its long-term success will depend on two things: technological credibility and substantial evidence. In practical terms, manufacturers must demonstrate that hydrogen is present at meaningful levels at the point of consumption, and researchers must clarify what “meaningful” entails—dose, frequency, and measurable outcomes.

Stability remains the main technical hurdle. Hydrogen can escape easily, and its retention depends heavily on the product matrix, packaging materials, storage time, and handling. While solutions for water use high-barrier packaging and related approaches, more complex products will require new strategies—such as dry formulations that generate hydrogen upon mixing or delivery systems designed to release hydrogen after ingestion. For the field to mature, it will also need better standards for measuring dissolved hydrogen, for reporting processing conditions, and for linking product quality to biological effects in a reproducible manner.
